# Supplementary figures and images for: aiSEGcell: User-friendly deep learning-based segmentation of nuclei in transmitted light images
Source: PLoS Comput Biol. 2024 Aug 23;20(8):e1012361. doi: 10.1371/journal.pcbi.1012361 (PMC11343410; doi:10.1371/journal.pcbi.1012361)

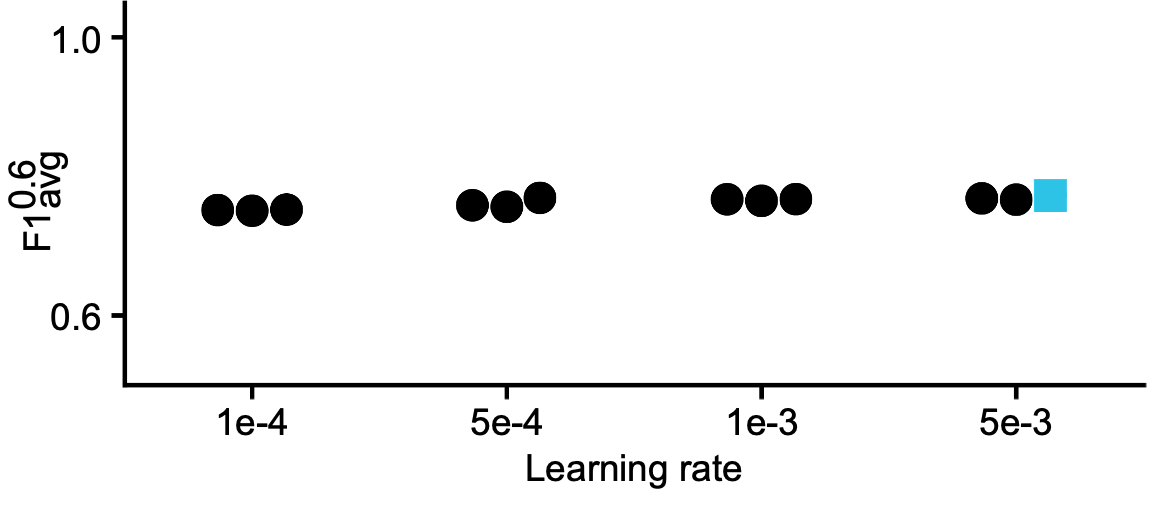

Supplement: S1 Fig — aiSEGcell instances trained with different learning rates on the D1 training set were evaluated with respect to F1avg0.6 on the D1 test set (n = 3,153 images, N = 12 experiments). All other hyperparameters were identical between hyperparameter conditions. For each learning rate three random weight initializations were trained. The instance with the highest F1avg0.6 (cyan square) was used for all subsequent experiments. (TIF) [file pcbi.1012361.s001.tif]

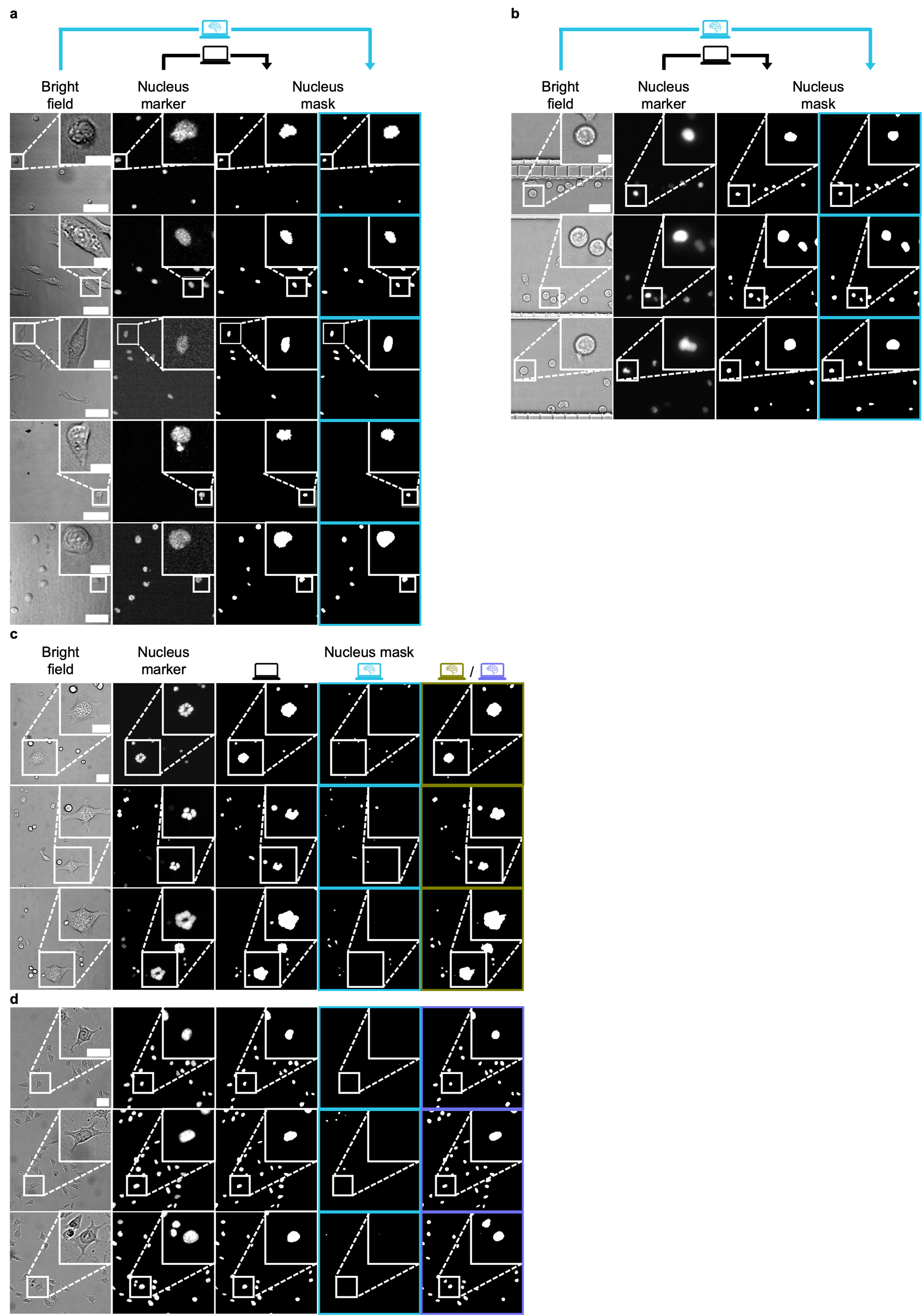

Supplement: S2 Fig — Nuclear segmentations in bright field were qualitatively similar to fluorescent nuclear marker derived segmentations. Images were cropped from a larger field-of-view for visibility and stem from a different independent experiment of (a) D2 (scale bar large images 40 μm, zoom ins 10 μm), (b) D5 (scale bar large image 40 μm, zoom in 10 μm), (c) D3 (scale bars 40 μm), and (d) D4 (scale bar large image 40 μm, zoom in 30 μm). Contrast individually adjusted here to improve visibility. Computers represent ground truth (black), aiSEGcell trained on D1 (cyan), aiSEGcell pretrained on D1 and retrained on D3 (green), and aiSEGcell pretrained on D1 and retrained on D4 (purple). (TIF) [file pcbi.1012361.s002.tif]

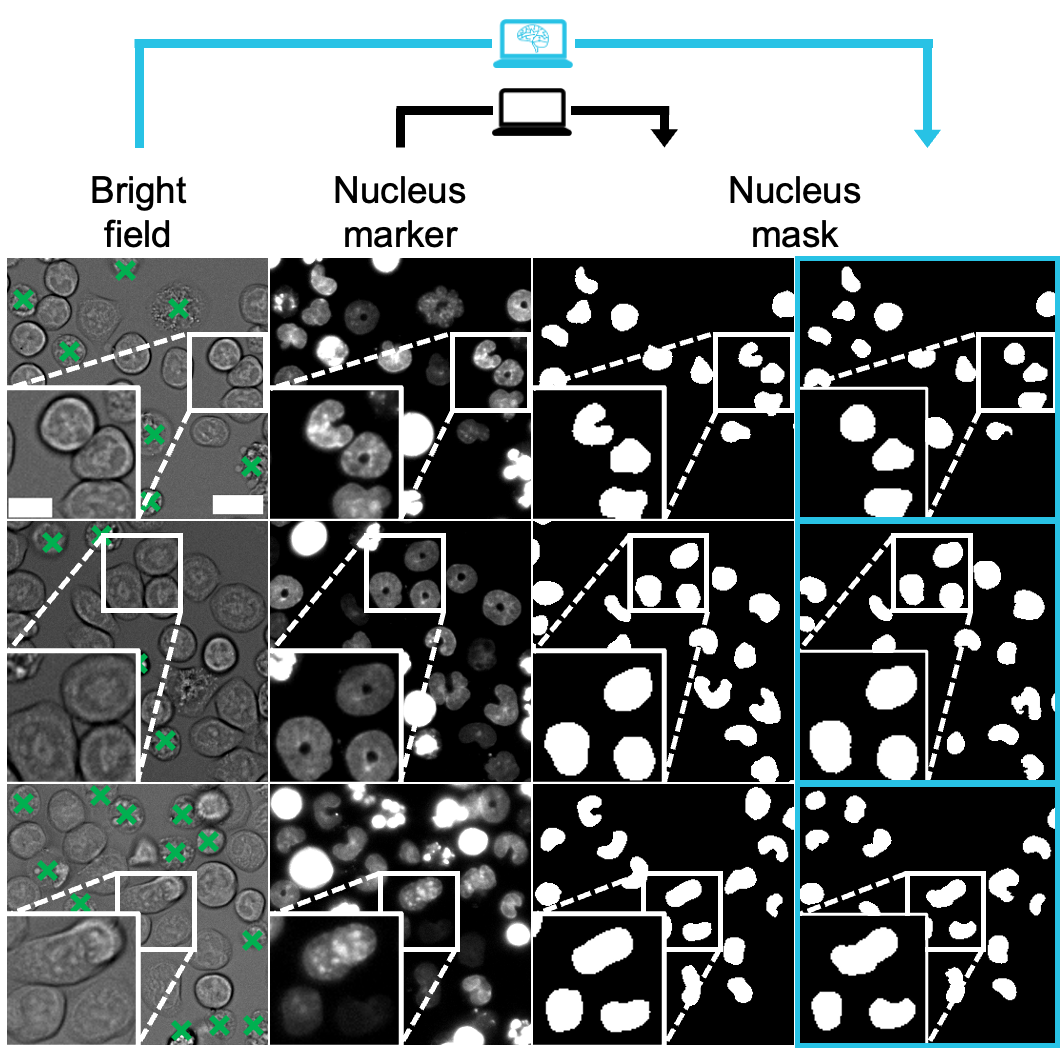

Supplement: S3 Fig — Nuclear segmentations in bright field were qualitatively similar to fluorescent nuclear marker derived segmentations. Images were cropped from a larger field-of-view (scale bar large image 20 μm, zoom in 10 μm). Contrast individually adjusted here to improve visibility. Computers represent ground truth (black) and aiSEGcell trained on D1 (cyan). Dead cells are crossed-out in green. (TIF) [file pcbi.1012361.s003.tif]

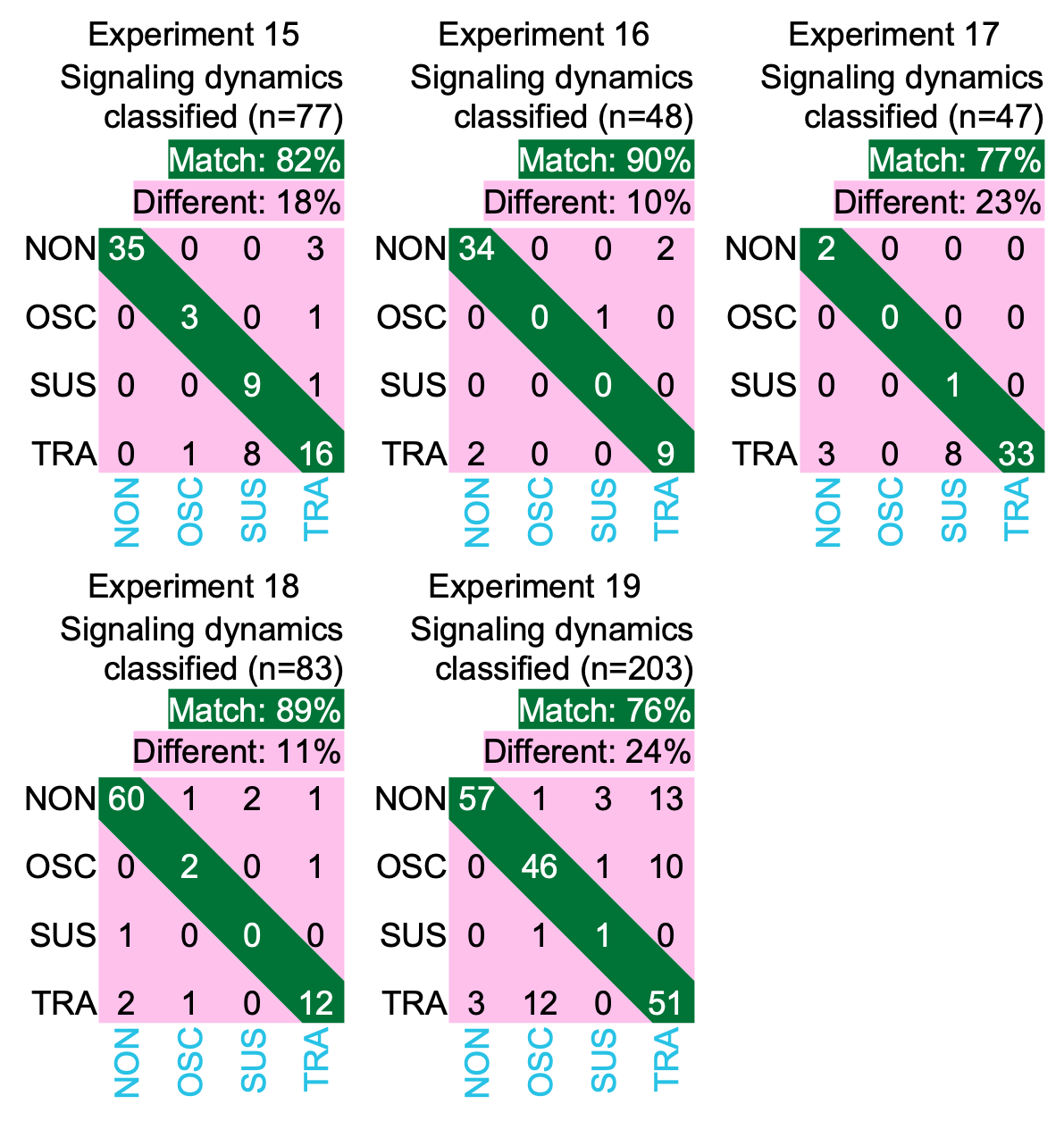

Supplement: S4 Fig — Signaling dynamics are classified into four response types: non-responsive (NON), oscillatory (OSC), sustained (SUS), and transient (TRA). Signaling dynamics were derived from D1-trained aiSEGcell nuclear segmentations (cyan) and manually curated ground truth segmentations (black). Experiment identifiers are taken from S3 Table (experiment 15 / 16 / 17 / 18 / 19 n = 77 / 48 / 47 / 83 / 203 signaling dynamics). (TIF) [file pcbi.1012361.s004.tif]

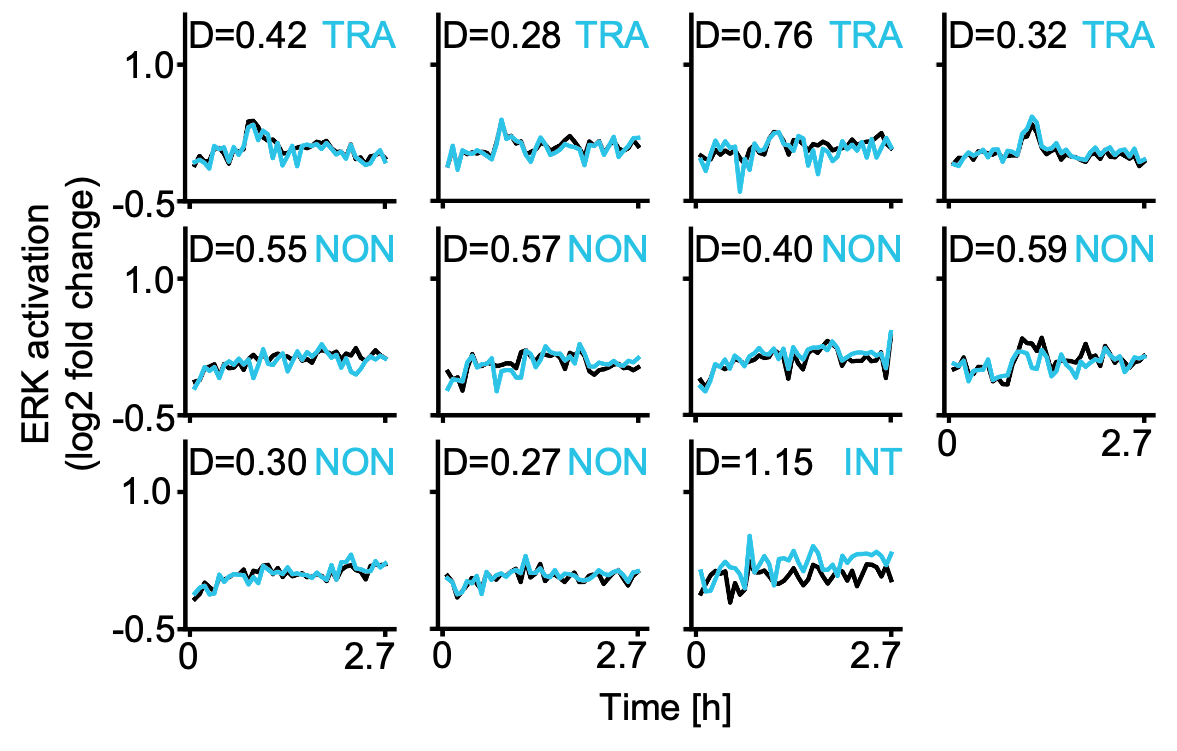

Supplement: S5 Fig — Manually curated ground truth (black) signaling dynamics of D5 were compared to the corresponding signaling dynamics based on D1-trained aiSEGcell (cyan) segmentations. All shown representative examples were classified as transient (TRA) from ground truth traces. The classification based on aiSEGcell segmentation is shown in cyan in each graph. Ground truth and aiSEGcell based signaling dynamics curves are very similar and would not be manually classified differently by an expert user, except maybe the last shown case. Euclidean distance (D) illustrates the high similarity between aiSEGcell and ground truth signaling dynamics. List of abbreviations: non-responsive (NON), intermediate (INT). (TIF) [file pcbi.1012361.s005.tif]

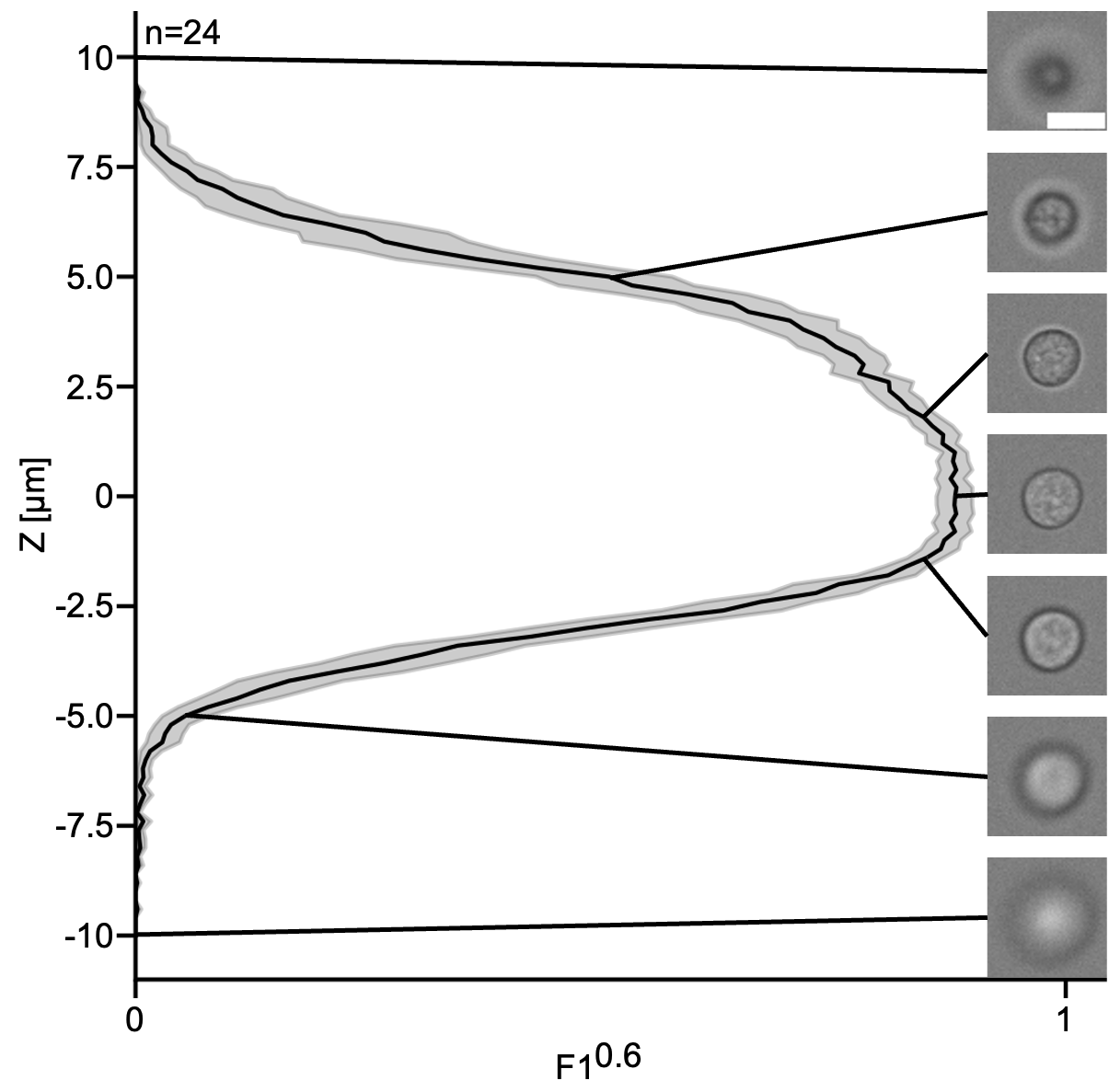

Supplement: S6 Fig — F1avg0.6 (black line) for 24 different field-of-views (N = 1 biological replicate, gray area is 95% confidence interval). Z of 0 corresponds to the optimal imaging plane selected by the experimenter. Z offsets were acquired with a step-size of 0.2 μm. Images from +10 μm, +5 μm, +1.8 μm, 0 μm, -1.4 μm, -5 μm, and -10 μm are shown here. Images were cropped from a larger field-of-view and contrast was individually adjusted here to improve visibility (scale bar 10 μm). (TIF) [file pcbi.1012361.s006.tif]

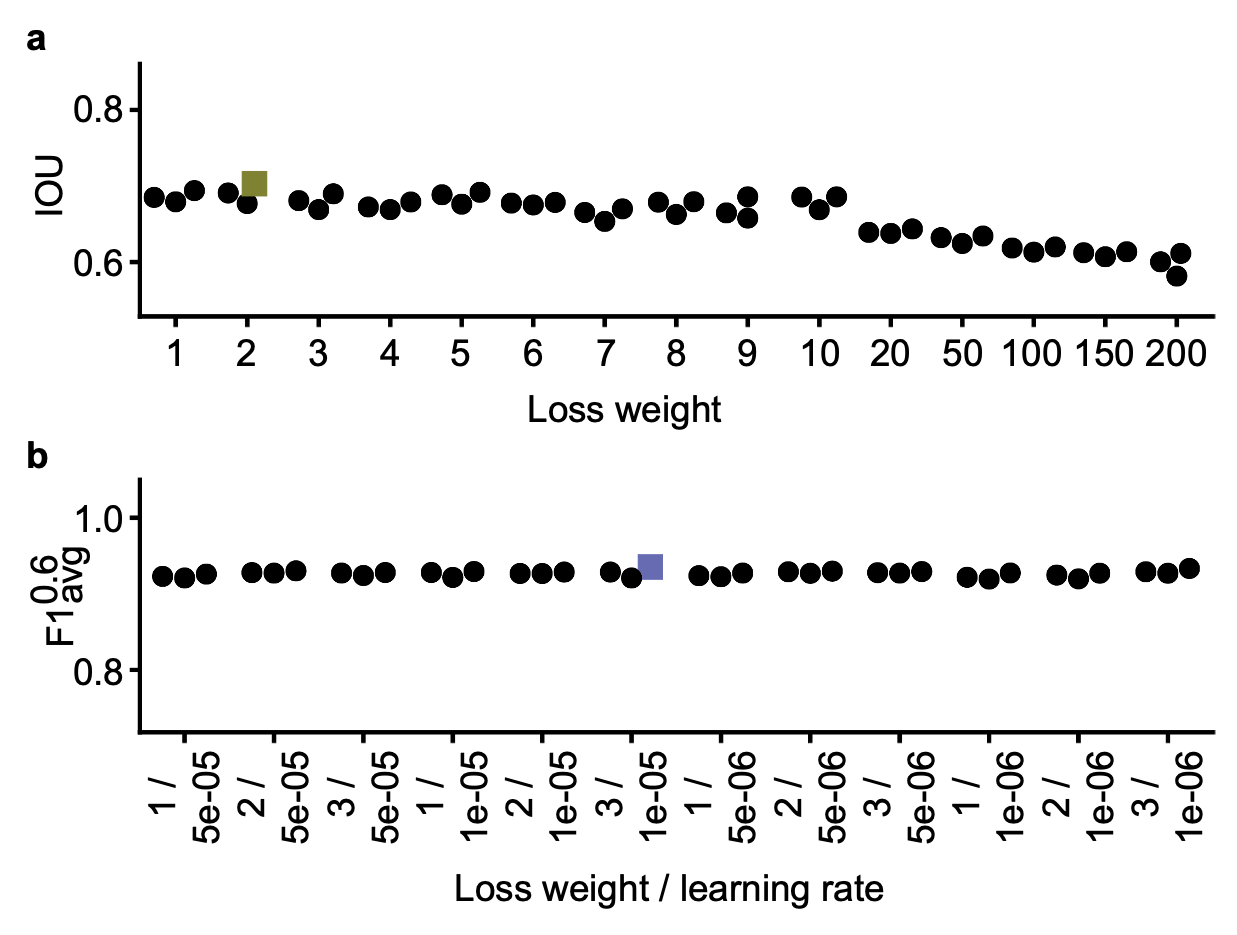

Supplement: S7 Fig — D1 pretrained aiSEGcell, retrained on (a) D3 (hyperparameter: binary cross entropy loss weights; evaluation: average image-wise intersection over union (IOU)) and (b) D4 (hyperparameters: binary cross entropy loss weights, learning rate; evaluation: F1avg0.6). All other hyperparameters were identical between hyperparameter conditions. For each hyperparameter combination three random weight initializations were trained. Models were evaluated on the respective validation set (colored square is best model; n = 10 unique field of views, N = 1 biological replicate). (TIF) [file pcbi.1012361.s007.tif]

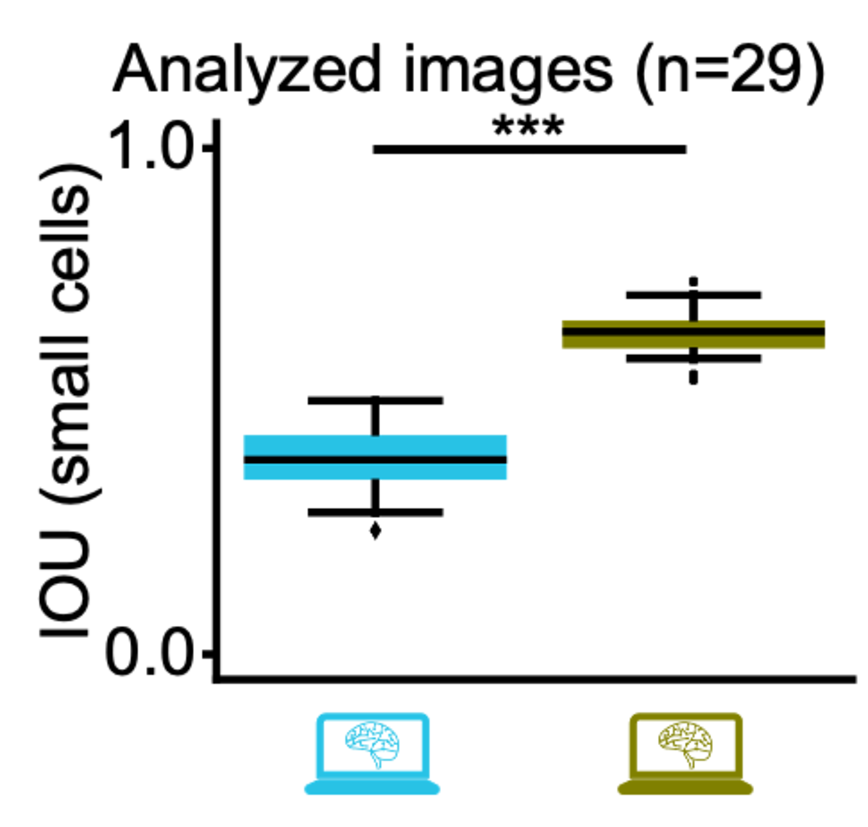

Supplement: S8 Fig — Average image-wise small cell (nuclei mask≤750 μm2) intersection over union (IOU) of the model pretrained on D1 (murine macrophages, hematopoietic stem- and progenitor cells; cyan; mean = 0.38, s.d. = 0.06) compared to the same model retrained on D3 (murine megakaryocytes, and hematopoietic stem cells; green; mean = 0.64, s.d. = 0.04). Compared to the analysis of big cells, 8 additional images that exclusively contained small cells were included (n = 29 unique field of views, N = 1 biological replicate; two-sided paired t-test, degrees of freedom = 28, t = -20.8, p = 1.4e-18; ***: p<0.001). In boxplots, the central black line is median, box boundaries are upper and lower quartiles, whiskers are 1.5x interquartile range. (TIF) [file pcbi.1012361.s008.tif]

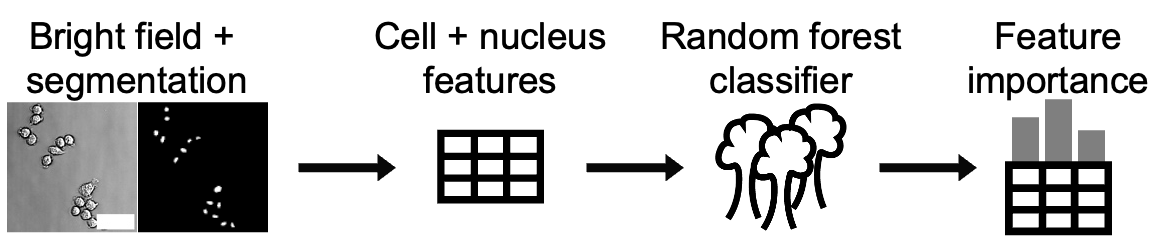

Supplement: S9 Fig — Individual cells were selected from D2 (999 high intersection over union (IOU) cells, 951 low IOU cells). Based on ground truth segmentations 21 features were computed for each cell, that described the morphology or intensity of nucleus, whole cell, or cell neighborhood. The random forest classifier with the best set of hyperparameters was trained (and validated) on 200 cells to distinguish between accurately and poorly segmented cells in a test set of 200 cells. Data sets were balanced and randomly compiled. Feature importance (measured by Gini impurity) was derived from the trained random forest classifier. Images were cropped from a larger field-of-view and contrast was individually adjusted here to improve visibility (scale bar 40 μm). (TIF) [file pcbi.1012361.s009.tif]

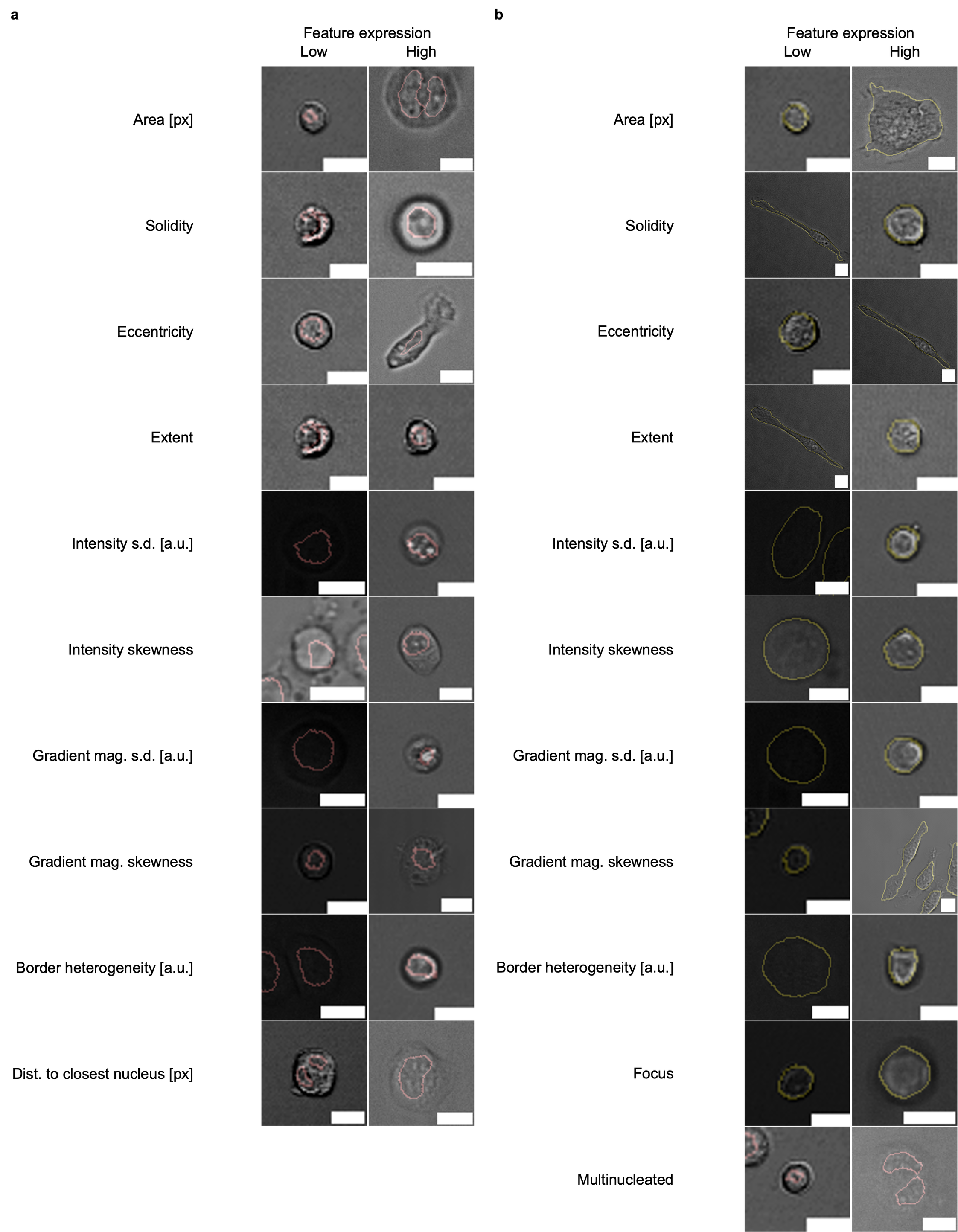

Supplement: S10 Fig — For each cell (a) 10 nucleus features and (b) 11 whole cell features were computed. For each feature, an example cell that has a high (High) or a low (Low) expression of the respective feature is shown. Images were cropped from a larger field-of-view and contrast was individually adjusted to improve visibility here for shape-based features only (Area, Solidity, Eccentricity, Extent, Distance to closest nucleus, Multinucleated). Contour of nucleus mask in red, contour of whole cell mask in yellow (scale bars 10 μm). (TIF) [file pcbi.1012361.s010.tif]

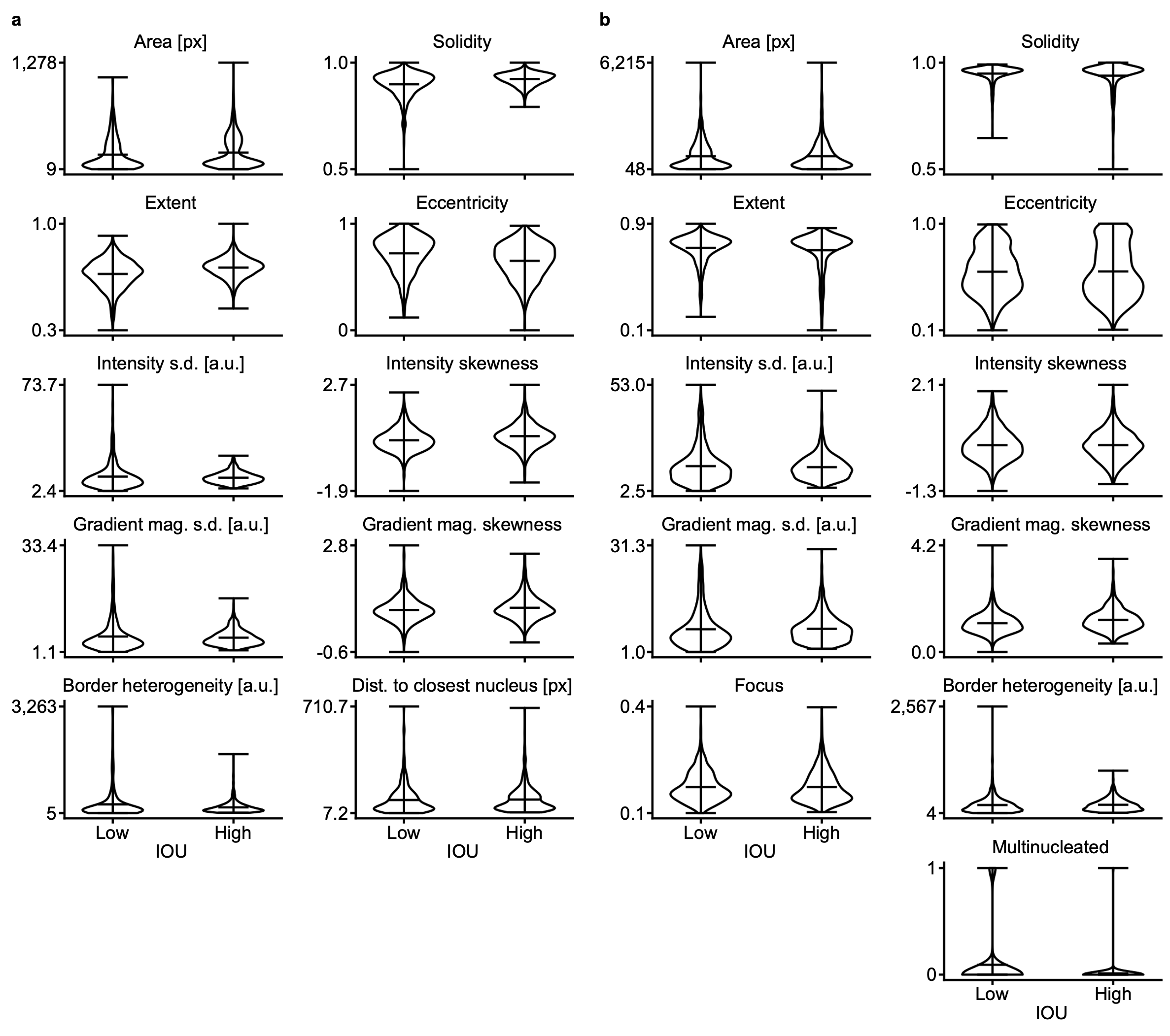

Supplement: S11 Fig — Distribution of (a) 10 nucleus features and (b) 11 whole cell features for high intersection over union (IOU; High; n = 999) and low IOU (Low, n = 951) segmented cells. For violin plots, whiskers depict the minimum and maximum, the central black line is the mean. (TIF) [file pcbi.1012361.s011.tif]

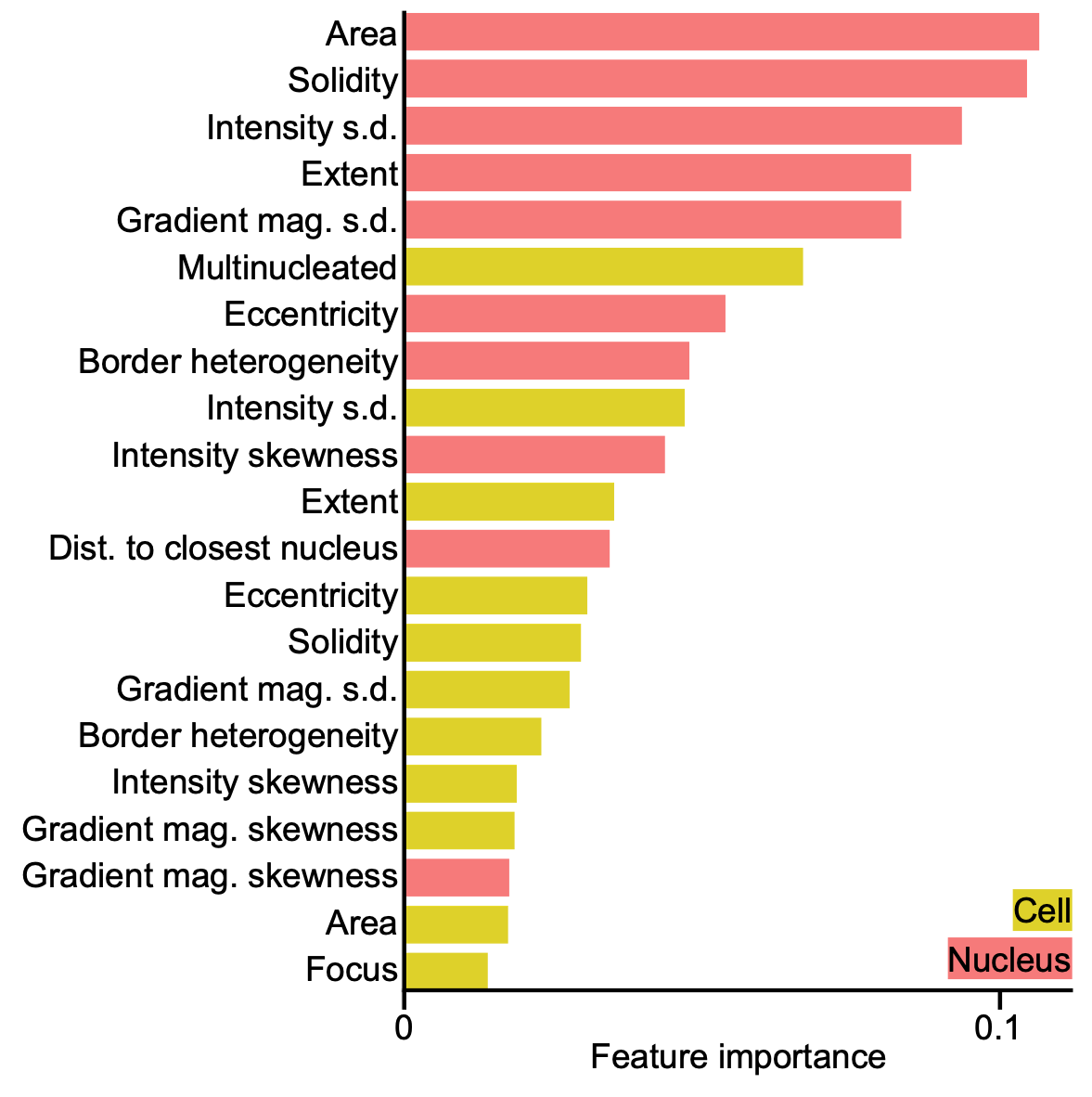

Supplement: S12 Fig — Feature importance (measured by Gini impurity; larger value means feature is more important) of the 21 features derived from the trained random forest classifier. Features were computed for the whole cell (yellow) or the nucleus (red). (TIF) [file pcbi.1012361.s012.tif]

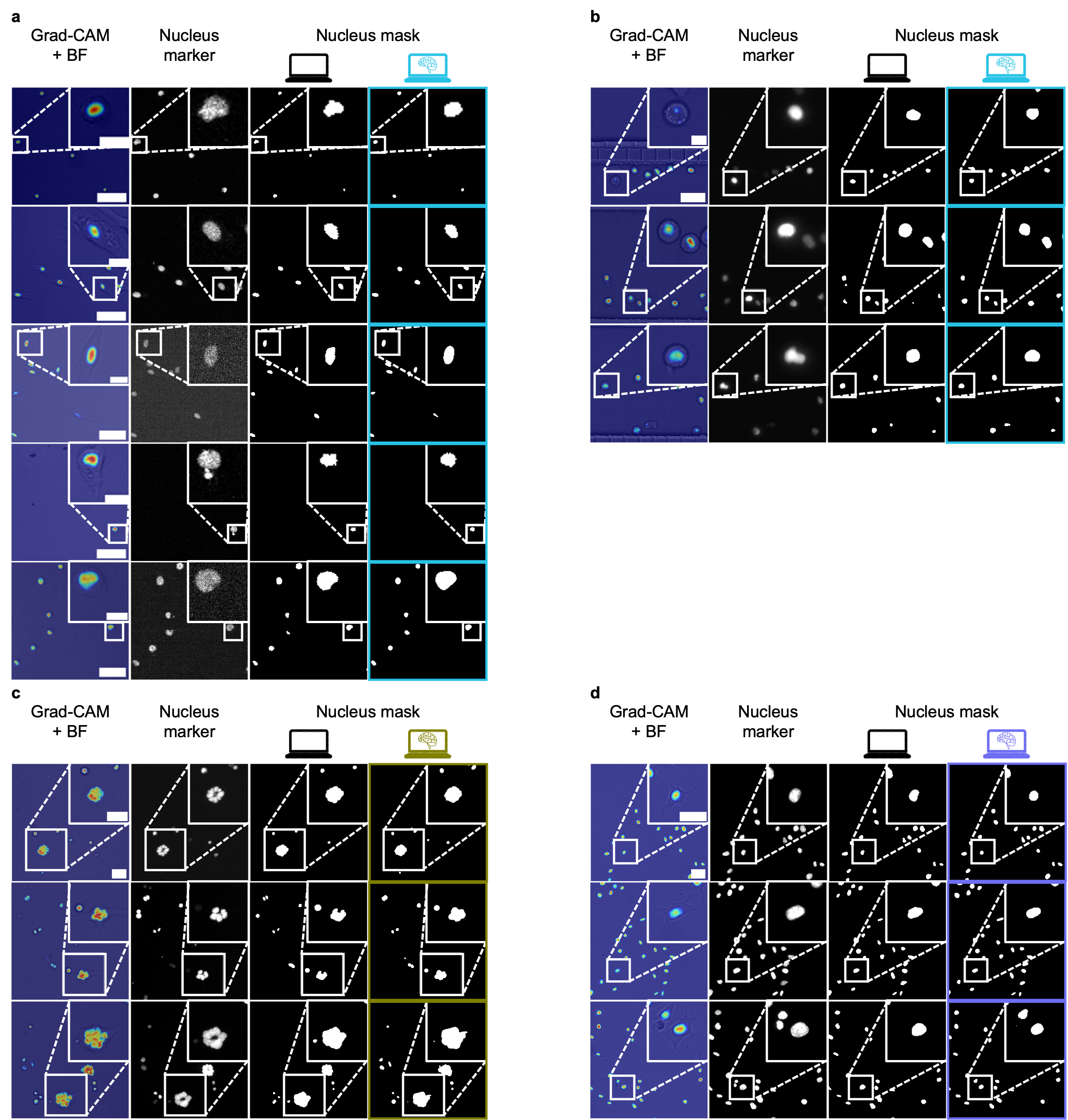

Supplement: S13 Fig — Grad-CAM of images depicted (a) in Figs 1c and S2a (scale bar large images 40 μm, zoom ins 10 μm), (b) in Figs 3a and S2b (scale bar large image 40 μm, zoom in 10 μm), (c) in Figs 3e and S2c (scale bar large image 40 μm, zoom in 40 μm), and (d) in Figs 3g and S2d (scale bar large image 40 μm, zoom in 30 μm). For bright field (BF) and nucleus marker images the contrast was individually adjusted to improve visibility and Grad-CAM color maps were overlayed. aiSEGcell was trained on D1 (cyan) or retrained on D3 (green) and D4 (purple) after D1 pretraining. In Grad-CAM color maps, colors indicate weak (blue) and strong (red) contributions of pixels to segmented objects. (TIF) [file pcbi.1012361.s013.tif]

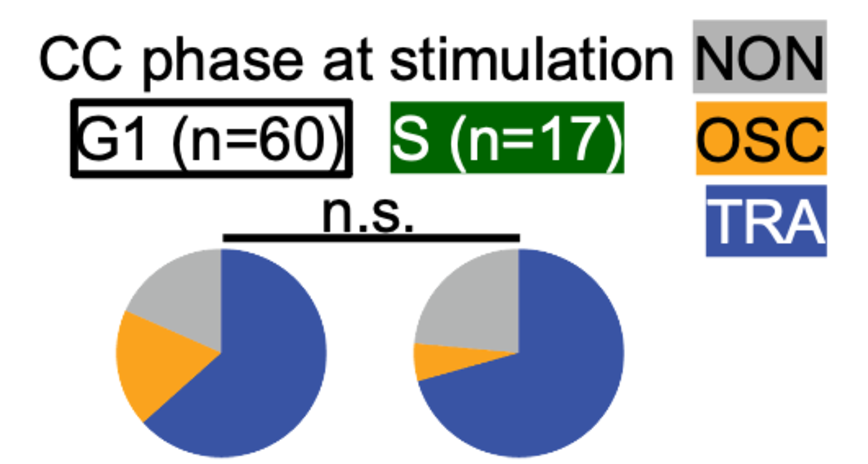

Supplement: S14 Fig — Only signaling dynamics of mGMPGM stimulated with TNFα are shown. One outlier cell tree was removed due to weak PCNA signal (n = 77 cell trees, N = 2 biological replicates; one-sided Chi2 test, degrees of freedom = 16, Chi2 statistic = 1.8, p = 0.401; n.s.: p≥0.05). List of abbreviations: cell cycle (CC), non-responsive (NON), oscillatory (OSC), transient (TRA). (TIF) [file pcbi.1012361.s014.tif]

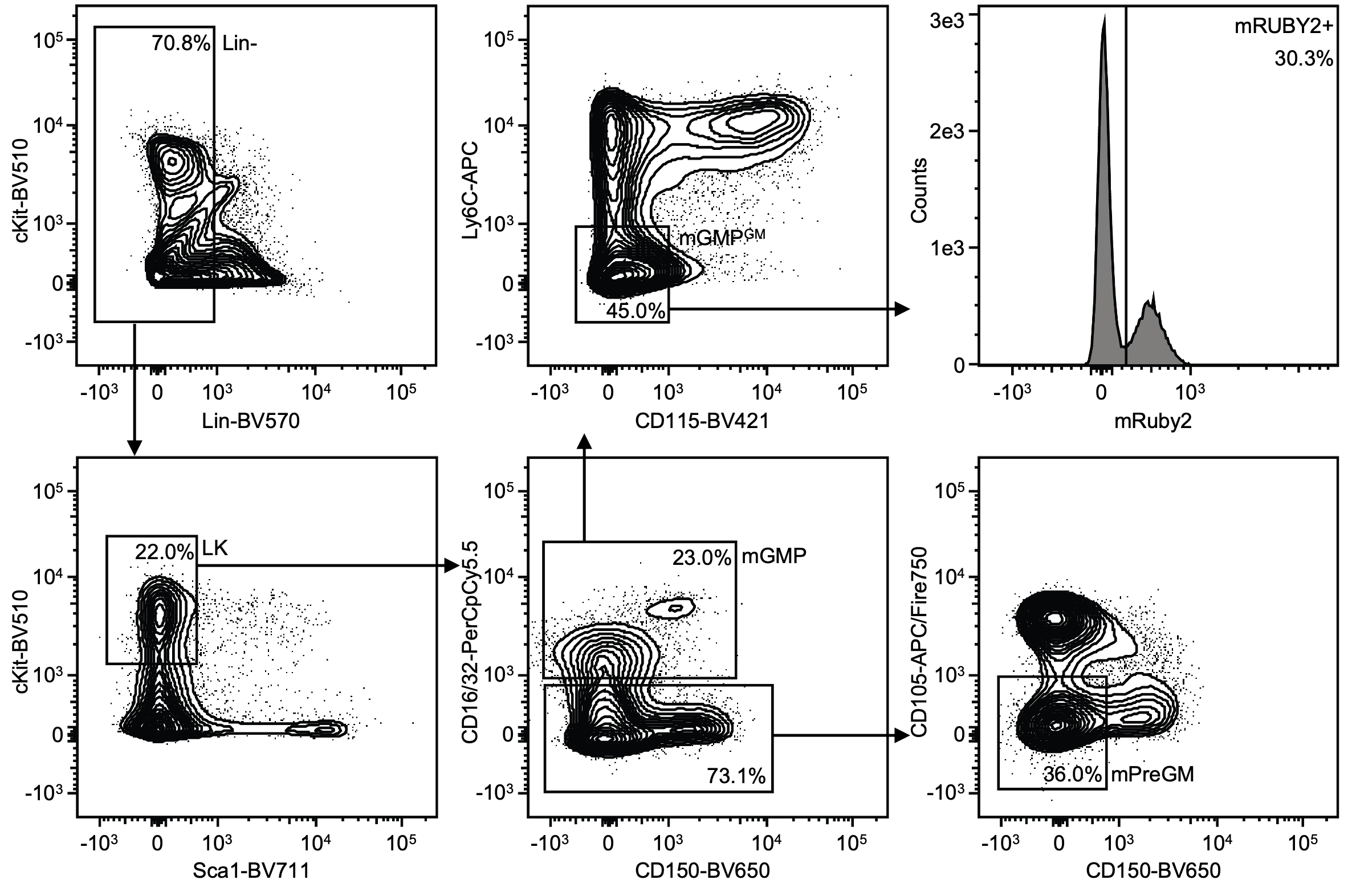

Supplement: S15 Fig — Isolation strategy for murine pre-granulocyte/monocyte progenitors (mPreGM, first sort) and murine lineage unbiased granulocyte/monocyte progenitor cells (mGMPGM, second sort). List of abbreviations: lineage negative (Lin-), lineage negative cKit positive (LK), murine granulocyte/monocyte progenitor (mGMP), monomeric RUBY2 positive (mRUBY2+). (TIF) [file pcbi.1012361.s015.tif]

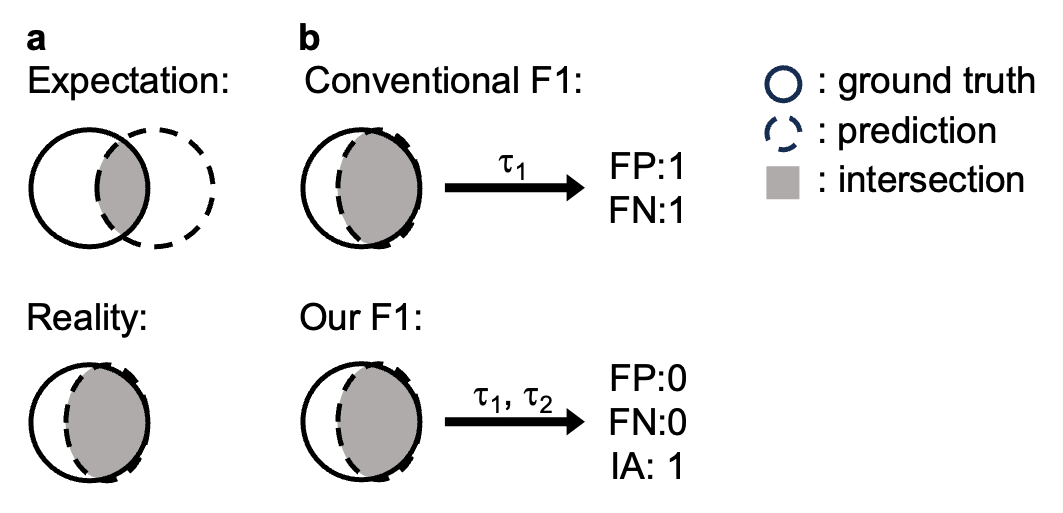

Supplement: S16 Fig — (a) Inaccurately predicted segmentations that only partially cover the ground truth segmentation are very common in reality. Despite what schematic representations often suggest, predictions that cover an area that does not correspond to a ground truth object (= false positive) rarely partially overlap with ground truth segmentations. (b) The introduction of a second threshold τ2 allows counting this common inaccurate mask error (IA) and prevents counting it as false positive (FP) and false negative (FN) error. (TIF) [file pcbi.1012361.s016.tif]
