# Supplementary material for: aiSEGcell: User-friendly deep learning-based segmentation of nuclei in transmitted light images
Source: PLoS Comput Biol. 2024 Aug 23;20(8):e1012361. doi: 10.1371/journal.pcbi.1012361 (PMC11343410; doi:10.1371/journal.pcbi.1012361)
Supplement: S2 Table — Scores in cells correspond to average conventional F1 +/- standard deviation (n = 3,153 images, N = 12 experiments) and τ1 refers to the intersection over union threshold above which predictions are considered true positives (best model per τ1 in bold). The cyan shaded row corresponds to the model we selected for testing and the cyan square in S1 Fig. List of abbreviations: StarDist (SD), Cellpose (CP), 2D_versatile_fluo model (Fluo), 2D_versatile_he model (HE), nuclei model (nuclei), yeast_BF_cp3 model (yeast BF), learning rate (lr), probability threshold (prob), non-maximum suppression threshold (nms), model-specific default (def), diameter (dia), flow threshold (flow). (DOCX) [file pcbi.1012361.s018.docx]

| Model | Hyperparameter | τ_1_=0.5 | τ_1_=0.55 | τ_1_=0.6 | τ_1_=0.65 | τ_1_=0.7 | τ_1_=0.75 | τ_1_=0.8 | τ_1_=0.85 | τ_1_=0.9 |
| --- | --- | --- | --- | --- | --- | --- | --- | --- | --- | --- |
| Ours | lr: 0.005 | 0.782  ±0.168 | 0.743  ±0.180 | 0.684  ±0.192 | 0.607  ±0.203 | 0.509  ±0.207 | 0.385  ±0.198 | 0.245  ±0.164 | 0.110  ±0.104 | 0.021  ±0.034 |
| Ours | lr: 0.001 | 0.780  ±0.169 | 0.739  ±0.180 | 0.682  ±0.193 | 0.609  ±0.203 | 0.509  ±0.206 | 0.383  ±0.194 | 0.239  ±0.154 | 0.101  ±0.092 | 0.019  ±0.029 |
| Ours | lr: 0.0005 | 0.772  ±0.179 | 0.730  ±0.189 | 0.671  ±0.199 | 0.594  ±0.207 | 0.493  ±0.209 | 0.368  ±0.195 | 0.230  ±0.155 | 0.100  ±0.093 | 0.019  ±0.031 |
| Ours | lr: 0.0001 | 0.770  ±0.183 | 0.727  ±0.197 | 0.666  ±0.209 | 0.588  ±0.219 | 0.487  ±0.220 | 0.363  ±0.202 | 0.226  ±0.160 | 0.099  ±0.095 | 0.018  ±0.030 |
| Ours | lr: 0.005 | 0.782  ±0.169 | 0.743  ±0.181 | 0.686  ±0.193 | 0.611  ±0.201 | 0.511  ±0.204 | 0.384  ±0.192 | 0.239  ±0.154 | 0.103  ±0.093 | 0.019  ±0.030 |
| Ours | lr: 0.001 | 0.783  ±0.175 | 0.743  ±0.188 | 0.684  ±0.202 | 0.609  ±0.213 | 0.510  ±0.216 | 0.385  ±0.203 | 0.243  ±0.165 | 0.108  ±0.105 | 0.021  ±0.034 |
| Ours | lr: 0.0005 | 0.776  ±0.174 | 0.734  ±0.184 | 0.673  ±0.197 | 0.597  ±0.207 | 0.495  ±0.211 | 0.369  ±0.198 | 0.229  ±0.156 | 0.098  ±0.094 | 0.018  ±0.030 |
| Ours | lr: 0.0001 | 0.768  ±0.181 | 0.726  ±0.195 | 0.666  ±0.208 | 0.589  ±0.217 | 0.492  ±0.215 | 0.368  ±0.199 | 0.228  ±0.156 | 0.097  ±0.091 | 0.017  ±0.028 |
| Ours | lr: 0.005 | **0.786**  **±0.171** | **0.749**  **±0.183** | **0.692**  **±0.198** | **0.619**  **±0.209** | **0.524**  **±0.215** | **0.401**  **±0.204** | **0.258**  **±0.167** | **0.116**  **±0.105** | 0.023  ±0.035 |
| Ours | lr: 0.001 | 0.782  ±0.172 | 0.742  ±0.184 | 0.683  ±0.198 | 0.608  ±0.210 | 0.511  ±0.214 | 0.388  ±0.203 | 0.250  ±0.167 | 0.114  ±0.105 | **0.023**  **±0.035** |
| Ours | lr: 0.0005 | 0.784  ±0.172 | 0.744  ±0.185 | 0.686  ±0.197 | 0.610  ±0.203 | 0.510  ±0.204 | 0.382  ±0.189 | 0.236  ±0.150 | 0.099  ±0.091 | 0.018  ±0.031 |
| Ours | lr: 0.0001 | 0.766  ±0.181 | 0.724  ±0.194 | 0.663  ±0.206 | 0.587  ±0.214 | 0.487  ±0.215 | 0.362  ±0.199 | 0.223  ±0.156 | 0.095  ±0.090 | 0.016  ±0.028 |
| SD Fluo | prob: 0.5, nms: 0.2 | 0.049  ±0.072 | 0.029  ±0.046 | 0.016  ±0.029 | 0.009  ±0.019 | 0.004  ±0.012 | 0.002  ±0.007 | 0.000  ±0.003 | 0.000  ±0.001 | 0.000  ±0.000 |
| SD Fluo | prob: 0.5, nms: 0.4 | 0.049  ±0.072 | 0.029  ±0.046 | 0.016  ±0.029 | 0.009  ±0.019 | 0.004  ±0.012 | 0.002  ±0.007 | 0.000  ±0.003 | 0.000  ±0.001 | 0.000  ±0.000 |
| SD Fluo | prob: 0.5, nms: def | 0.049  ±0.072 | 0.029  ±0.046 | 0.016  ±0.029 | 0.009  ±0.019 | 0.004  ±0.012 | 0.002  ±0.007 | 0.000  ±0.003 | 0.000  ±0.001 | 0.000  ±0.000 |
| SD Fluo | prob: 0.8, nms: 0.2 | 0.030  ±0.066 | 0.016  ±0.044 | 0.008  ±0.028 | 0.004  ±0.020 | 0.002  ±0.013 | 0.001  ±0.007 | 0.000  ±0.003 | 0.000  ±0.000 | 0.000  ±0.000 |
| SD Fluo | prob: 0.8, nms: 0.4 | 0.030  ±0.066 | 0.016  ±0.044 | 0.008  ±0.028 | 0.004  ±0.020 | 0.002  ±0.013 | 0.001  ±0.007 | 0.000  ±0.003 | 0.000  ±0.000 | 0.000  ±0.000 |
| SD Fluo | prob: 0.8, nms: def | 0.030  ±0.066 | 0.016  ±0.044 | 0.008  ±0.028 | 0.004  ±0.020 | 0.002  ±0.013 | 0.001  ±0.007 | 0.000  ±0.003 | 0.000  ±0.000 | 0.000  ±0.000 |
| SD Fluo | prob: def, nms: 0.2 | 0.050  ±0.072 | 0.029  ±0.046 | 0.016  ±0.029 | 0.009  ±0.019 | 0.004  ±0.012 | 0.002  ±0.007 | 0.001  ±0.003 | 0.000  ±0.001 | 0.000  ±0.000 |
| SD Fluo | prob: def, nms: 0.4 | 0.049  ±0.072 | 0.029  ±0.046 | 0.016  ±0.029 | 0.009  ±0.019 | 0.004  ±0.012 | 0.002  ±0.007 | 0.001  ±0.003 | 0.000  ±0.001 | 0.000  ±0.000 |
| SD Fluo | prob: def, nms: def | 0.050  ±0.072 | 0.029  ±0.046 | 0.016  ±0.029 | 0.009  ±0.019 | 0.004  ±0.012 | 0.002  ±0.007 | 0.001  ±0.003 | 0.000  ±0.001 | 0.000  ±0.000 |
| SD HE | prob: 0.5, nms: 0.2 | 0.012  ±0.027 | 0.007  ±0.018 | 0.004  ±0.012 | 0.002  ±0.007 | 0.001  ±0.004 | 0.000  ±0.003 | 0.000  ±0.002 | 0.000  ±0.001 | 0.000  ±0.000 |
| SD HE | prob: 0.5, nms: 0.4 | 0.012  ±0.027 | 0.007  ±0.018 | 0.004  ±0.012 | 0.002  ±0.007 | 0.001  ±0.004 | 0.000  ±0.003 | 0.000  ±0.001 | 0.000  ±0.001 | 0.000  ±0.000 |
| SD HE | prob: 0.5, nms: def | 0.012  ±0.027 | 0.007  ±0.018 | 0.004  ±0.012 | 0.002  ±0.007 | 0.001  ±0.004 | 0.000  ±0.003 | 0.000  ±0.001 | 0.000  ±0.001 | 0.000  ±0.000 |
| SD HE | prob: 0.8, nms: 0.2 | 0.005  ±0.018 | 0.003  ±0.011 | 0.002  ±0.009 | 0.001  ±0.005 | 0.000  ±0.002 | 0.000  ±0.001 | 0.000  ±0.000 | 0.000  ±0.000 | 0.000  ±0.000 |
| SD HE | prob: 0.8, nms: 0.4 | 0.005  ±0.018 | 0.003  ±0.011 | 0.002  ±0.009 | 0.001  ±0.005 | 0.000  ±0.002 | 0.000  ±0.001 | 0.000  ±0.000 | 0.000  ±0.000 | 0.000  ±0.000 |
| SD HE | prob: 0.8, nms: def | 0.005  ±0.018 | 0.003  ±0.011 | 0.002  ±0.009 | 0.001  ±0.005 | 0.000  ±0.002 | 0.000  ±0.001 | 0.000  ±0.000 | 0.000  ±0.000 | 0.000  ±0.000 |
| SD HE | prob: def, nms: 0.2 | 0.009  ±0.024 | 0.005  ±0.016 | 0.003  ±0.011 | 0.001  ±0.006 | 0.001  ±0.004 | 0.000  ±0.002 | 0.000  ±0.001 | 0.000  ±0.001 | 0.000  ±0.000 |
| SD HE | prob: def, nms: 0.4 | 0.009  ±0.024 | 0.005  ±0.016 | 0.003  ±0.011 | 0.001  ±0.006 | 0.001  ±0.004 | 0.000  ±0.002 | 0.000  ±0.001 | 0.000  ±0.001 | 0.000  ±0.000 |
| SD HE | prob: def, nms: def | 0.009  ±0.024 | 0.005  ±0.016 | 0.003  ±0.011 | 0.001  ±0.006 | 0.001  ±0.004 | 0.000  ±0.002 | 0.000  ±0.001 | 0.000  ±0.001 | 0.000  ±0.000 |
| CP nuclei | dia: 17, flow: 0.2 | 0.080  ±0.111 | 0.052  ±0.080 | 0.032  ±0.056 | 0.019  ±0.040 | 0.010  ±0.026 | 0.005  ±0.015 | 0.002  ±0.009 | 0.001  ±0.004 | 0.000  ±0.001 |
| CP nuclei | dia: 17, flow: 0.4 | 0.096  ±0.112 | 0.063  ±0.078 | 0.038  ±0.053 | 0.021  ±0.035 | 0.011  ±0.022 | 0.005  ±0.013 | 0.002  ±0.008 | 0.001  ±0.004 | 0.000  ±0.001 |
| CP nuclei | dia: 17, flow: 0.6 | 0.099  ±0.111 | 0.064  ±0.077 | 0.038  ±0.051 | 0.021  ±0.033 | 0.010  ±0.020 | 0.004  ±0.011 | 0.002  ±0.007 | 0.000  ±0.003 | 0.000  ±0.001 |
| CP nuclei | dia: 30, flow: 0.2 | 0.085  ±0.121 | 0.055  ±0.085 | 0.032  ±0.055 | 0.019  ±0.039 | 0.011  ±0.027 | 0.006  ±0.017 | 0.003  ±0.011 | 0.001  ±0.006 | 0.000  ±0.002 |
| CP nuclei | dia: 30, flow: 0.4 | 0.120  ±0.117 | 0.082  ±0.085 | 0.051  ±0.060 | 0.032  ±0.044 | 0.019  ±0.031 | 0.009  ±0.019 | 0.004  ±0.011 | 0.001  ±0.005 | 0.000  ±0.002 |
| CP nuclei | dia: 30, flow: 0.6 | 0.128  ±0.116 | 0.086  ±0.085 | 0.054  ±0.060 | 0.032  ±0.042 | 0.018  ±0.028 | 0.008  ±0.017 | 0.003  ±0.009 | 0.001  ±0.004 | 0.000  ±0.001 |
| CP nuclei | dia: 40, flow: 0.2 | 0.067  ±0.128 | 0.041  ±0.088 | 0.021  ±0.057 | 0.011  ±0.038 | 0.006  ±0.025 | 0.003  ±0.015 | 0.001  ±0.009 | 0.000  ±0.004 | 0.000  ±0.002 |
| CP nuclei | dia: 40, flow: 0.4 | 0.101  ±0.123 | 0.064  ±0.087 | 0.037  ±0.060 | 0.021  ±0.040 | 0.011  ±0.027 | 0.005  ±0.016 | 0.002  ±0.009 | 0.000  ±0.004 | 0.000  ±0.001 |
| CP nuclei | dia: 40, flow: 0.6 | 0.114  ±0.119 | 0.073  ±0.085 | 0.042  ±0.059 | 0.023  ±0.039 | 0.012  ±0.024 | 0.005  ±0.013 | 0.002  ±0.007 | 0.000  ±0.003 | 0.000  ±0.001 |
| CP yeast BF | dia: 17, flow: 0.2 | 0.034  ±0.078 | 0.021  ±0.054 | 0.012  ±0.035 | 0.007  ±0.021 | 0.003  ±0.013 | 0.001  ±0.007 | 0.001  ±0.004 | 0.000  ±0.002 | 0.000  ±0.000 |
| CP yeast BF | dia: 17, flow: 0.4 | 0.042  ±0.078 | 0.025  ±0.053 | 0.014  ±0.033 | 0.007  ±0.020 | 0.003  ±0.012 | 0.001  ±0.006 | 0.000  ±0.004 | 0.000  ±0.002 | 0.000  ±0.000 |
| CP yeast BF | dia: 17, flow: 0.6 | 0.040  ±0.076 | 0.024  ±0.051 | 0.013  ±0.032 | 0.007  ±0.019 | 0.003  ±0.011 | 0.001  ±0.006 | 0.000  ±0.003 | 0.000  ±0.002 | 0.000  ±0.000 |
| CP yeast BF | dia: 30, flow: 0.2 | 0.048  ±0.099 | 0.031  ±0.072 | 0.019  ±0.048 | 0.011  ±0.029 | 0.005  ±0.015 | 0.002  ±0.009 | 0.001  ±0.005 | 0.000  ±0.002 | 0.000  ±0.000 |
| CP yeast BF | dia: 30, flow: 0.4 | 0.053  ±0.098 | 0.034  ±0.071 | 0.020  ±0.047 | 0.011  ±0.028 | 0.005  ±0.015 | 0.002  ±0.008 | 0.001  ±0.005 | 0.000  ±0.002 | 0.000  ±0.000 |
| CP yeast BF | dia: 30, flow: 0.6 | 0.053  ±0.096 | 0.034  ±0.070 | 0.020  ±0.046 | 0.011  ±0.028 | 0.005  ±0.014 | 0.002  ±0.008 | 0.001  ±0.005 | 0.000  ±0.002 | 0.000  ±0.000 |
| CP yeast BF | dia: 40, flow: 0.2 | 0.057  ±0.106 | 0.037  ±0.078 | 0.023  ±0.055 | 0.014  ±0.037 | 0.008  ±0.023 | 0.004  ±0.015 | 0.002  ±0.009 | 0.000  ±0.004 | 0.000  ±0.002 |
| CP yeast BF | dia: 40, flow: 0.4 | 0.066  ±0.109 | 0.043  ±0.080 | 0.027  ±0.057 | 0.016  ±0.038 | 0.008  ±0.023 | 0.004  ±0.015 | 0.002  ±0.008 | 0.000  ±0.003 | 0.000  ±0.002 |
| CP yeast BF | dia: 40, flow: 0.6 | 0.068  ±0.107 | 0.044  ±0.079 | 0.027  ±0.056 | 0.016  ±0.038 | 0.008  ±0.023 | 0.004  ±0.014 | 0.002  ±0.008 | 0.000  ±0.003 | 0.000  ±0.002 |

S2 Table: Conventional F1-scores for the D1 test set.

Scores in cells correspond to average conventional F1 +/- standard deviation (n=3,153 images, N=12 experiments) and τ_1_ refers to the intersection over union threshold above which predictions are considered true positives (best model per τ_1_ in bold). The cyan shaded row corresponds to the model we selected for testing and the cyan square in S1 Fig. List of abbreviations: StarDist (SD), Cellpose (CP), 2D_versatile_fluo model (Fluo), 2D_versatile_he model (HE), nuclei model (nuclei), yeast_BF_cp3 model (yeast BF), learning rate (lr), probability threshold (prob), non-maximum suppression threshold (nms), model-specific default (def), diameter (dia), flow threshold (flow).
