# Supplementary material for: aiSEGcell: User-friendly deep learning-based segmentation of nuclei in transmitted light images
Source: PLoS Comput Biol. 2024 Aug 23;20(8):e1012361. doi: 10.1371/journal.pcbi.1012361 (PMC11343410; doi:10.1371/journal.pcbi.1012361)
Supplement: S4 Table — The average (avg) refers to the average of image-wise computed performance metrics. The aggregate (agg) of performance metrics refers to a data set-wise computation. Data set D7 refers to the higher density experiments. All metrics were acquired using τ1 = 0.6 and τ2 = 0.1. List of abbreviations: true positive (TP), false negative (FN), false positive (FP), intersection over union (IOU). (DOCX) [file pcbi.1012361.s020.docx]

| **Data set** | D2 | D3 | D4 | D5 | D5 curated | D7 |
| --- | --- | --- | --- | --- | --- | --- |
| **TP avg** | 26.88 | 24 | 249.1 | 4.47 | 5.9 | 123.4 |
| **TP agg** | 167,782 | 696 | 2,491 | 3,650 | 4,817 | 1,234 |
| **FN avg** | 3.94 | 1.07 | 5.5 | 1.89 | 0.54 | 22.1 |
| **FN agg** | 24,579 | 31 | 55 | 1,542 | 438 | 221 |
| **FP avg** | 4.91 | 14.93 | 19.1 | 4.28 | 1.77 | 26.5 |
| **FP agg** | 30,669 | 433 | 191 | 3,490 | 1,441 | 265 |
| **Inaccurate masks avg** | 18.33 | 4.55 | 15.8 | 5.26 | 5.14 | 14.1 |
| **Inaccurate masks agg** | 114,449 | 132 | 158 | 4,291 | 4,198 | 141 |
| **Splits avg** | 0.2 | 0 | 0 | 0.01 | 0 | 0.5 |
| **Splits agg** | 1,260 | 0 | 0 | 7 | 3 | 5 |
| **Merges avg** | 0.24 | 2.07 | 0.4 | 0 | 0.06 | 0.7 |
| **Merges agg** | 1,514 | 60 | 4 | 0 | 50 | 7 |
| $\text{F1}_{\text{avg}}^{\text{0.6}}$ | 0.66 | 0.69 | 0.93 | 0.45 | 0.62 | 0.80 |
| $\text{F1}_{\mathbf{agg}}^{\text{0.6}}$ | 0.66 | 0.70 | 0.92 | 0.44 | 0.61 | 0.80 |
| **IOU small** | X | 0.64 | 0.75 | x | x | x |
| **IOU big** | X | 0.77 | x | x | x | x |
| **IOU all** | X | 0.69 | 0.75 | x | x | x |
| **N** | 6243 | 29 | 10 | 816 | 816 | 10 |

S4 Table: Test set performance for all data sets.

The average (avg) refers to the average of image-wise computed performance metrics. The aggregate (agg) of performance metrics refers to a data set-wise computation. Data set D7 refers to the higher density experiments. All metrics were acquired using τ_1_=0.6 and τ_2_=0.1. List of abbreviations: true positive (TP), false negative (FN), false positive (FP), intersection over union (IOU).
