# Supplementary material for: aiSEGcell: User-friendly deep learning-based segmentation of nuclei in transmitted light images
Source: PLoS Comput Biol. 2024 Aug 23;20(8):e1012361. doi: 10.1371/journal.pcbi.1012361 (PMC11343410; doi:10.1371/journal.pcbi.1012361)
Supplement: S5 Table — Scores in cells correspond to average conventional or adapted F1 +/- standard deviation on the higher density experiments data set D7 (n = 10 images, N = 3 experiments) and τ1 refers to the intersection over union threshold above which predictions are considered true positives. D1 trained model (cyan) corresponds to the respectively colored square in S1 Fig. (DOCX) [file pcbi.1012361.s021.docx]

| Model | Score | τ_1_=0.5 | τ_1_=0.55 | τ_1_=0.6 | τ_1_=0.65 | τ_1_=0.7 | τ_1_=0.75 | τ_1_=0.8 | τ_1_=0.85 | τ_1_=0.9 |
| --- | --- | --- | --- | --- | --- | --- | --- | --- | --- | --- |
| D1 trained | adapted | 0.822 ±0.033 | 0.813 ±0.032 | 0.797 ±0.034 | 0.762 ±0.033 | 0.708 ±0.044 | 0.617 ±0.063 | 0.486 ±0.085 | 0.290 ±0.079 | 0.078 ±0.044 |
| D1 trained | conventional | 0.795 ±0.037 | 0.780 ±0.036 | 0.755 ±0.038 | 0.702 ±0.037 | 0.626 ±0.055 | 0.511 ±0.073 | 0.370 ±0.082 | 0.195 ±0.059 | 0.047 ±0.027 |

S5 Table: F1-scores for D7 test set.

Scores in cells correspond to average conventional or adapted F1 +/- standard deviation on the higher density experiments data set D7 (n=10 images, N=3 experiments) and τ_1_ refers to the intersection over union threshold above which predictions are considered true positives. D1 trained model (cyan) corresponds to the respectively colored square in S1 Fig.
