# Supplementary material for: aiSEGcell: User-friendly deep learning-based segmentation of nuclei in transmitted light images
Source: PLoS Comput Biol. 2024 Aug 23;20(8):e1012361. doi: 10.1371/journal.pcbi.1012361 (PMC11343410; doi:10.1371/journal.pcbi.1012361)
Supplement: S6 Table — Scores in cells correspond to average conventional or adapted F1 +/- standard deviation (n = 816 images, N = 1 experiment) and τ1 refers to the intersection over union threshold above which predictions are considered true positives. D1 trained model (cyan) corresponds to the respectively colored square in S1 Fig. (DOCX) [file pcbi.1012361.s022.docx]

| Model | Score | Curated | τ_1_=0.5 | τ_1_=0.55 | τ_1_=0.6 | τ_1_=0.65 | τ_1_=0.7 | τ_1_=0.75 | τ_1_=0.8 | τ_1_=0.85 | τ_1_=0.9 |
| --- | --- | --- | --- | --- | --- | --- | --- | --- | --- | --- | --- |
| D1 trained | adapted | y | 0.766 ±0.158 | 0.707 ±0.169 | 0.620 ±0.181 | 0.497 ±0.200 | 0.348 ±0.209 | 0.207 ±0.184 | 0.090 ±0.146 | 0.024 ±0.075 | 0.004 ±0.033 |
| D1 trained | conventional | y | 0.700 ±0.176 | 0.621 ±0.186 | 0.516 ±0.192 | 0.386 ±0.192 | 0.252 ±0.182 | 0.140 ±0.148 | 0.060 ±0.114 | 0.015 ±0.049 | 0.003 ±0.021 |
| D1 trained | adapted | n | 0.566 ±0.189 | 0.517 ±0.193 | 0.450 ±0.194 | 0.361 ±0.187 | 0.256 ±0.175 | 0.154 ±0.149 | 0.068 ±0.114 | 0.020 ±0.065 | 0.004 ±0.028 |
| D1 trained | conventional | n | 0.506 ±0.189 | 0.450 ±0.190 | 0.377 ±0.185 | 0.288 ±0.167 | 0.193 ±0.146 | 0.111 ±0.116 | 0.048 ±0.085 | 0.014 ±0.046 | 0.002 ±0.019 |

S6 Table: F1-scores for the D5 test set.

Scores in cells correspond to average conventional or adapted F1 +/- standard deviation (n=816 images, N=1 experiment) and τ_1_ refers to the intersection over union threshold above which predictions are considered true positives. D1 trained model (cyan) corresponds to the respectively colored square in S1 Fig.
