# Supplementary material for: aiSEGcell: User-friendly deep learning-based segmentation of nuclei in transmitted light images
Source: PLoS Comput Biol. 2024 Aug 23;20(8):e1012361. doi: 10.1371/journal.pcbi.1012361 (PMC11343410; doi:10.1371/journal.pcbi.1012361)
Supplement: S7 Table — Scores in cells correspond to average adapted F1 +/- standard deviation (n = 24 images, N = 1 experiment) and τ1 refers to the intersection over union threshold above which predictions are considered true positives. Analysis corresponds to S6 Fig and rows shaded in grey mark z-layers -1.8 μm to +2.6 μm. (DOCX) [file pcbi.1012361.s023.docx]

| Focal plane [μm] | τ_1_=0.5 | τ_1_=0.55 | τ_1_=0.6 | τ_1_=0.65 | τ_1_=0.7 | τ_1_=0.75 | τ_1_=0.8 | τ_1_=0.85 | τ_1_=0.9 |
| --- | --- | --- | --- | --- | --- | --- | --- | --- | --- |
| -10 | 0.002 ±0.008 | 0.002 ±0.008 | 0.000 ±0.000 | 0.000 ±0.000 | 0.000 ±0.000 | 0.000 ±0.000 | 0.000 ±0.000 | 0.000 ±0.000 | 0.000 ±0.000 |
| -9.8 | 0.000 ±0.000 | 0.000 ±0.000 | 0.000 ±0.000 | 0.000 ±0.000 | 0.000 ±0.000 | 0.000 ±0.000 | 0.000 ±0.000 | 0.000 ±0.000 | 0.000 ±0.000 |
| -9.6 | 0.000 ±0.000 | 0.000 ±0.000 | 0.000 ±0.000 | 0.000 ±0.000 | 0.000 ±0.000 | 0.000 ±0.000 | 0.000 ±0.000 | 0.000 ±0.000 | 0.000 ±0.000 |
| -9.4 | 0.002 ±0.010 | 0.002 ±0.010 | 0.002 ±0.010 | 0.000 ±0.000 | 0.000 ±0.000 | 0.000 ±0.000 | 0.000 ±0.000 | 0.000 ±0.000 | 0.000 ±0.000 |
| -9.2 | 0.000 ±0.000 | 0.000 ±0.000 | 0.000 ±0.000 | 0.000 ±0.000 | 0.000 ±0.000 | 0.000 ±0.000 | 0.000 ±0.000 | 0.000 ±0.000 | 0.000 ±0.000 |
| -9 | 0.000 ±0.000 | 0.000 ±0.000 | 0.000 ±0.000 | 0.000 ±0.000 | 0.000 ±0.000 | 0.000 ±0.000 | 0.000 ±0.000 | 0.000 ±0.000 | 0.000 ±0.000 |
| -8.8 | 0.002 ±0.008 | 0.002 ±0.008 | 0.002 ±0.008 | 0.000 ±0.000 | 0.000 ±0.000 | 0.000 ±0.000 | 0.000 ±0.000 | 0.000 ±0.000 | 0.000 ±0.000 |
| -8.6 | 0.002 ±0.008 | 0.000 ±0.000 | 0.000 ±0.000 | 0.000 ±0.000 | 0.000 ±0.000 | 0.000 ±0.000 | 0.000 ±0.000 | 0.000 ±0.000 | 0.000 ±0.000 |
| -8.4 | 0.007 ±0.016 | 0.005 ±0.015 | 0.003 ±0.011 | 0.002 ±0.009 | 0.000 ±0.000 | 0.000 ±0.000 | 0.000 ±0.000 | 0.000 ±0.000 | 0.000 ±0.000 |
| -8.2 | 0.009 ±0.021 | 0.003 ±0.011 | 0.002 ±0.008 | 0.002 ±0.008 | 0.002 ±0.008 | 0.000 ±0.000 | 0.000 ±0.000 | 0.000 ±0.000 | 0.000 ±0.000 |
| -8 | 0.005 ±0.014 | 0.005 ±0.014 | 0.005 ±0.014 | 0.005 ±0.014 | 0.005 ±0.014 | 0.002 ±0.008 | 0.000 ±0.000 | 0.000 ±0.000 | 0.000 ±0.000 |
| -7.8 | 0.006 ±0.019 | 0.006 ±0.019 | 0.004 ±0.014 | 0.003 ±0.012 | 0.000 ±0.000 | 0.000 ±0.000 | 0.000 ±0.000 | 0.000 ±0.000 | 0.000 ±0.000 |
| -7.6 | 0.012 ±0.025 | 0.012 ±0.025 | 0.003 ±0.011 | 0.003 ±0.011 | 0.002 ±0.008 | 0.002 ±0.008 | 0.002 ±0.008 | 0.002 ±0.008 | 0.000 ±0.000 |
| -7.4 | 0.010 ±0.021 | 0.008 ±0.020 | 0.008 ±0.020 | 0.005 ±0.014 | 0.002 ±0.008 | 0.000 ±0.000 | 0.000 ±0.000 | 0.000 ±0.000 | 0.000 ±0.000 |
| -7.2 | 0.005 ±0.014 | 0.004 ±0.012 | 0.002 ±0.008 | 0.002 ±0.008 | 0.002 ±0.008 | 0.000 ±0.000 | 0.000 ±0.000 | 0.000 ±0.000 | 0.000 ±0.000 |
| -7 | 0.012 ±0.022 | 0.008 ±0.018 | 0.005 ±0.014 | 0.002 ±0.008 | 0.002 ±0.008 | 0.000 ±0.000 | 0.000 ±0.000 | 0.000 ±0.000 | 0.000 ±0.000 |
| -6.8 | 0.015 ±0.030 | 0.012 ±0.029 | 0.009 ±0.022 | 0.004 ±0.013 | 0.002 ±0.010 | 0.000 ±0.000 | 0.000 ±0.000 | 0.000 ±0.000 | 0.000 ±0.000 |
| -6.6 | 0.022 ±0.046 | 0.010 ±0.022 | 0.004 ±0.015 | 0.004 ±0.015 | 0.002 ±0.009 | 0.000 ±0.000 | 0.000 ±0.000 | 0.000 ±0.000 | 0.000 ±0.000 |
| -6.4 | 0.017 ±0.036 | 0.009 ±0.020 | 0.009 ±0.020 | 0.007 ±0.017 | 0.001 ±0.007 | 0.000 ±0.000 | 0.000 ±0.000 | 0.000 ±0.000 | 0.000 ±0.000 |
| -6.2 | 0.035 ±0.036 | 0.025 ±0.032 | 0.008 ±0.018 | 0.004 ±0.013 | 0.002 ±0.011 | 0.002 ±0.011 | 0.000 ±0.000 | 0.000 ±0.000 | 0.000 ±0.000 |
| -6 | 0.026 ±0.042 | 0.017 ±0.029 | 0.011 ±0.020 | 0.009 ±0.019 | 0.004 ±0.014 | 0.000 ±0.000 | 0.000 ±0.000 | 0.000 ±0.000 | 0.000 ±0.000 |
| -5.8 | 0.036 ±0.067 | 0.022 ±0.044 | 0.016 ±0.031 | 0.012 ±0.023 | 0.009 ±0.019 | 0.006 ±0.016 | 0.000 ±0.000 | 0.000 ±0.000 | 0.000 ±0.000 |
| -5.6 | 0.066 ±0.074 | 0.041 ±0.061 | 0.029 ±0.045 | 0.009 ±0.025 | 0.003 ±0.011 | 0.003 ±0.011 | 0.002 ±0.008 | 0.000 ±0.000 | 0.000 ±0.000 |
| -5.4 | 0.071 ±0.091 | 0.049 ±0.068 | 0.032 ±0.048 | 0.019 ±0.035 | 0.009 ±0.026 | 0.003 ±0.011 | 0.000 ±0.000 | 0.000 ±0.000 | 0.000 ±0.000 |
| -5.2 | 0.104 ±0.118 | 0.065 ±0.083 | 0.038 ±0.049 | 0.021 ±0.043 | 0.014 ±0.032 | 0.006 ±0.019 | 0.001 ±0.007 | 0.001 ±0.007 | 0.000 ±0.000 |
| -5 | 0.127 ±0.126 | 0.088 ±0.108 | 0.053 ±0.065 | 0.040 ±0.051 | 0.023 ±0.035 | 0.005 ±0.013 | 0.001 ±0.007 | 0.001 ±0.007 | 0.000 ±0.000 |
| -4.8 | 0.156 ±0.140 | 0.113 ±0.118 | 0.078 ±0.088 | 0.036 ±0.056 | 0.023 ±0.037 | 0.013 ±0.028 | 0.001 ±0.007 | 0.000 ±0.000 | 0.000 ±0.000 |
| -4.6 | 0.203 ±0.161 | 0.162 ±0.138 | 0.109 ±0.109 | 0.062 ±0.076 | 0.031 ±0.055 | 0.017 ±0.038 | 0.009 ±0.022 | 0.003 ±0.011 | 0.000 ±0.000 |
| -4.4 | 0.241 ±0.164 | 0.180 ±0.160 | 0.134 ±0.129 | 0.086 ±0.093 | 0.047 ±0.065 | 0.021 ±0.043 | 0.012 ±0.031 | 0.003 ±0.010 | 0.000 ±0.000 |
| -4.2 | 0.312 ±0.200 | 0.248 ±0.169 | 0.165 ±0.142 | 0.109 ±0.112 | 0.050 ±0.070 | 0.024 ±0.053 | 0.007 ±0.023 | 0.002 ±0.008 | 0.002 ±0.008 |
| -4 | 0.354 ±0.230 | 0.289 ±0.205 | 0.214 ±0.165 | 0.147 ±0.126 | 0.083 ±0.077 | 0.034 ±0.050 | 0.009 ±0.022 | 0.000 ±0.000 | 0.000 ±0.000 |
| -3.8 | 0.411 ±0.215 | 0.335 ±0.203 | 0.266 ±0.180 | 0.193 ±0.154 | 0.107 ±0.102 | 0.066 ±0.070 | 0.027 ±0.038 | 0.008 ±0.022 | 0.000 ±0.000 |
| -3.6 | 0.454 ±0.230 | 0.388 ±0.218 | 0.309 ±0.193 | 0.214 ±0.151 | 0.148 ±0.121 | 0.080 ±0.074 | 0.031 ±0.037 | 0.010 ±0.024 | 0.000 ±0.000 |
| -3.4 | 0.514 ±0.214 | 0.445 ±0.202 | 0.346 ±0.186 | 0.252 ±0.162 | 0.167 ±0.135 | 0.085 ±0.089 | 0.038 ±0.056 | 0.011 ±0.022 | 0.000 ±0.000 |
| -3.2 | 0.601 ±0.168 | 0.515 ±0.180 | 0.423 ±0.178 | 0.311 ±0.178 | 0.208 ±0.152 | 0.116 ±0.108 | 0.055 ±0.063 | 0.012 ±0.023 | 0.003 ±0.012 |
| -3 | 0.657 ±0.161 | 0.579 ±0.171 | 0.485 ±0.178 | 0.368 ±0.178 | 0.247 ±0.160 | 0.162 ±0.141 | 0.075 ±0.081 | 0.019 ±0.048 | 0.000 ±0.000 |
| -2.8 | 0.731 ±0.139 | 0.657 ±0.143 | 0.552 ±0.166 | 0.426 ±0.173 | 0.319 ±0.147 | 0.190 ±0.129 | 0.090 ±0.090 | 0.020 ±0.030 | 0.000 ±0.000 |
| -2.6 | 0.771 ±0.124 | 0.713 ±0.150 | 0.632 ±0.164 | 0.515 ±0.153 | 0.365 ±0.156 | 0.223 ±0.119 | 0.097 ±0.082 | 0.033 ±0.046 | 0.003 ±0.015 |
| -2.4 | 0.809 ±0.106 | 0.763 ±0.119 | 0.672 ±0.156 | 0.560 ±0.152 | 0.434 ±0.148 | 0.273 ±0.133 | 0.137 ±0.099 | 0.049 ±0.048 | 0.007 ±0.015 |
| -2.2 | 0.833 ±0.094 | 0.793 ±0.108 | 0.732 ±0.124 | 0.639 ±0.124 | 0.508 ±0.121 | 0.342 ±0.138 | 0.158 ±0.100 | 0.063 ±0.070 | 0.008 ±0.023 |
| -2 | 0.869 ±0.064 | 0.830 ±0.080 | 0.756 ±0.127 | 0.665 ±0.134 | 0.544 ±0.135 | 0.390 ±0.122 | 0.192 ±0.112 | 0.067 ±0.084 | 0.011 ±0.022 |
| -1.8 | 0.885 ±0.050 | 0.857 ±0.054 | 0.809 ±0.083 | 0.732 ±0.111 | 0.627 ±0.119 | 0.453 ±0.117 | 0.232 ±0.108 | 0.082 ±0.084 | 0.016 ±0.035 |
| -1.6 | 0.894 ±0.048 | 0.875 ±0.049 | 0.828 ±0.058 | 0.757 ±0.094 | 0.658 ±0.114 | 0.489 ±0.127 | 0.282 ±0.120 | 0.117 ±0.079 | 0.019 ±0.032 |
| -1.4 | 0.895 ±0.041 | 0.881 ±0.041 | 0.850 ±0.049 | 0.786 ±0.083 | 0.695 ±0.106 | 0.542 ±0.124 | 0.319 ±0.111 | 0.128 ±0.095 | 0.024 ±0.041 |
| -1.2 | 0.903 ±0.045 | 0.889 ±0.043 | 0.866 ±0.053 | 0.811 ±0.068 | 0.731 ±0.084 | 0.595 ±0.117 | 0.395 ±0.129 | 0.164 ±0.097 | 0.029 ±0.039 |
| -1 | 0.909 ±0.044 | 0.890 ±0.049 | 0.869 ±0.053 | 0.830 ±0.070 | 0.759 ±0.078 | 0.632 ±0.104 | 0.425 ±0.133 | 0.194 ±0.103 | 0.045 ±0.053 |
| -0.8 | 0.908 ±0.043 | 0.896 ±0.043 | 0.881 ±0.046 | 0.841 ±0.064 | 0.763 ±0.076 | 0.651 ±0.086 | 0.466 ±0.110 | 0.224 ±0.100 | 0.042 ±0.052 |
| -0.6 | 0.907 ±0.042 | 0.896 ±0.043 | 0.876 ±0.051 | 0.836 ±0.071 | 0.772 ±0.079 | 0.660 ±0.105 | 0.495 ±0.133 | 0.256 ±0.127 | 0.052 ±0.054 |
| -0.4 | 0.909 ±0.044 | 0.900 ±0.043 | 0.882 ±0.048 | 0.848 ±0.063 | 0.795 ±0.071 | 0.691 ±0.092 | 0.535 ±0.119 | 0.279 ±0.129 | 0.072 ±0.075 |
| -0.2 | 0.907 ±0.043 | 0.903 ±0.043 | 0.880 ±0.049 | 0.843 ±0.055 | 0.788 ±0.074 | 0.693 ±0.073 | 0.521 ±0.117 | 0.279 ±0.130 | 0.056 ±0.058 |
| 0 | 0.903 ±0.043 | 0.898 ±0.044 | 0.881 ±0.048 | 0.851 ±0.050 | 0.796 ±0.061 | 0.709 ±0.067 | 0.555 ±0.090 | 0.304 ±0.109 | 0.077 ±0.073 |
| 0.2 | 0.904 ±0.039 | 0.897 ±0.041 | 0.882 ±0.044 | 0.848 ±0.047 | 0.795 ±0.061 | 0.715 ±0.064 | 0.543 ±0.103 | 0.305 ±0.106 | 0.063 ±0.051 |
| 0.4 | 0.899 ±0.038 | 0.895 ±0.038 | 0.876 ±0.041 | 0.847 ±0.049 | 0.790 ±0.056 | 0.701 ±0.070 | 0.528 ±0.101 | 0.287 ±0.100 | 0.064 ±0.061 |
| 0.6 | 0.903 ±0.039 | 0.897 ±0.040 | 0.882 ±0.043 | 0.842 ±0.056 | 0.780 ±0.066 | 0.703 ±0.057 | 0.532 ±0.095 | 0.281 ±0.130 | 0.043 ±0.055 |
| 0.8 | 0.904 ±0.038 | 0.896 ±0.038 | 0.879 ±0.043 | 0.842 ±0.051 | 0.788 ±0.055 | 0.686 ±0.061 | 0.521 ±0.083 | 0.275 ±0.100 | 0.057 ±0.048 |
| 1 | 0.903 ±0.032 | 0.896 ±0.033 | 0.881 ±0.035 | 0.841 ±0.054 | 0.777 ±0.059 | 0.666 ±0.058 | 0.498 ±0.087 | 0.255 ±0.113 | 0.049 ±0.061 |
| 1.2 | 0.898 ±0.037 | 0.888 ±0.037 | 0.868 ±0.041 | 0.824 ±0.054 | 0.745 ±0.054 | 0.633 ±0.063 | 0.452 ±0.093 | 0.240 ±0.109 | 0.055 ±0.048 |
| 1.4 | 0.904 ±0.039 | 0.894 ±0.044 | 0.868 ±0.047 | 0.827 ±0.055 | 0.747 ±0.054 | 0.616 ±0.090 | 0.422 ±0.105 | 0.205 ±0.099 | 0.031 ±0.037 |
| 1.6 | 0.893 ±0.040 | 0.882 ±0.041 | 0.857 ±0.051 | 0.796 ±0.062 | 0.717 ±0.079 | 0.584 ±0.082 | 0.403 ±0.106 | 0.177 ±0.107 | 0.027 ±0.047 |
| 1.8 | 0.894 ±0.036 | 0.880 ±0.039 | 0.847 ±0.048 | 0.794 ±0.074 | 0.689 ±0.086 | 0.577 ±0.098 | 0.378 ±0.125 | 0.136 ±0.077 | 0.023 ±0.030 |
| 2 | 0.890 ±0.038 | 0.870 ±0.046 | 0.831 ±0.055 | 0.774 ±0.071 | 0.680 ±0.083 | 0.556 ±0.121 | 0.333 ±0.142 | 0.135 ±0.098 | 0.022 ±0.032 |
| 2.2 | 0.889 ±0.045 | 0.868 ±0.050 | 0.822 ±0.061 | 0.764 ±0.077 | 0.661 ±0.100 | 0.504 ±0.119 | 0.313 ±0.118 | 0.102 ±0.076 | 0.014 ±0.029 |
| 2.4 | 0.879 ±0.047 | 0.855 ±0.049 | 0.811 ±0.060 | 0.744 ±0.076 | 0.632 ±0.088 | 0.489 ±0.115 | 0.292 ±0.110 | 0.101 ±0.068 | 0.011 ±0.031 |
| 2.6 | 0.879 ±0.059 | 0.856 ±0.059 | 0.810 ±0.071 | 0.742 ±0.080 | 0.629 ±0.101 | 0.463 ±0.131 | 0.256 ±0.123 | 0.084 ±0.066 | 0.014 ±0.026 |
| 2.8 | 0.866 ±0.059 | 0.833 ±0.062 | 0.778 ±0.073 | 0.712 ±0.089 | 0.586 ±0.101 | 0.418 ±0.121 | 0.231 ±0.101 | 0.089 ±0.074 | 0.011 ±0.024 |
| 3 | 0.859 ±0.051 | 0.829 ±0.062 | 0.783 ±0.074 | 0.686 ±0.097 | 0.581 ±0.111 | 0.386 ±0.121 | 0.224 ±0.115 | 0.079 ±0.064 | 0.008 ±0.019 |
| 3.2 | 0.860 ±0.062 | 0.829 ±0.067 | 0.773 ±0.086 | 0.683 ±0.112 | 0.529 ±0.122 | 0.374 ±0.114 | 0.196 ±0.092 | 0.063 ±0.067 | 0.006 ±0.019 |
| 3.4 | 0.851 ±0.065 | 0.814 ±0.075 | 0.753 ±0.101 | 0.656 ±0.118 | 0.508 ±0.133 | 0.338 ±0.119 | 0.175 ±0.085 | 0.057 ±0.057 | 0.008 ±0.022 |
| 3.6 | 0.834 ±0.069 | 0.801 ±0.077 | 0.740 ±0.101 | 0.629 ±0.129 | 0.470 ±0.125 | 0.301 ±0.106 | 0.140 ±0.094 | 0.042 ±0.049 | 0.002 ±0.008 |
| 3.8 | 0.833 ±0.068 | 0.792 ±0.081 | 0.718 ±0.105 | 0.602 ±0.138 | 0.445 ±0.146 | 0.265 ±0.123 | 0.106 ±0.069 | 0.033 ±0.046 | 0.002 ±0.010 |
| 4 | 0.824 ±0.092 | 0.775 ±0.105 | 0.703 ±0.134 | 0.575 ±0.145 | 0.407 ±0.139 | 0.234 ±0.102 | 0.086 ±0.064 | 0.023 ±0.039 | 0.000 ±0.000 |
| 4.2 | 0.799 ±0.092 | 0.745 ±0.119 | 0.659 ±0.143 | 0.534 ±0.155 | 0.377 ±0.144 | 0.203 ±0.106 | 0.078 ±0.065 | 0.028 ±0.035 | 0.002 ±0.009 |
| 4.4 | 0.778 ±0.115 | 0.720 ±0.130 | 0.642 ±0.144 | 0.508 ±0.162 | 0.339 ±0.140 | 0.169 ±0.096 | 0.066 ±0.062 | 0.012 ±0.034 | 0.002 ±0.008 |
| 4.6 | 0.743 ±0.139 | 0.673 ±0.163 | 0.594 ±0.168 | 0.459 ±0.168 | 0.312 ±0.141 | 0.140 ±0.102 | 0.071 ±0.075 | 0.018 ±0.034 | 0.000 ±0.000 |
| 4.8 | 0.710 ±0.172 | 0.635 ±0.184 | 0.534 ±0.185 | 0.401 ±0.182 | 0.260 ±0.132 | 0.123 ±0.108 | 0.049 ±0.058 | 0.007 ±0.020 | 0.000 ±0.000 |
| 5 | 0.673 ±0.200 | 0.614 ±0.205 | 0.509 ±0.182 | 0.363 ±0.171 | 0.213 ±0.141 | 0.123 ±0.122 | 0.049 ±0.058 | 0.016 ±0.027 | 0.000 ±0.000 |
| 5.2 | 0.607 ±0.215 | 0.542 ±0.212 | 0.433 ±0.201 | 0.322 ±0.183 | 0.168 ±0.143 | 0.083 ±0.096 | 0.037 ±0.056 | 0.014 ±0.029 | 0.000 ±0.000 |
| 5.4 | 0.545 ±0.238 | 0.468 ±0.233 | 0.367 ±0.217 | 0.245 ±0.172 | 0.158 ±0.133 | 0.087 ±0.098 | 0.032 ±0.054 | 0.008 ±0.018 | 0.000 ±0.000 |
| 5.6 | 0.500 ±0.228 | 0.412 ±0.229 | 0.312 ±0.198 | 0.216 ±0.177 | 0.114 ±0.112 | 0.062 ±0.081 | 0.025 ±0.042 | 0.006 ±0.023 | 0.000 ±0.000 |
| 5.8 | 0.438 ±0.258 | 0.337 ±0.232 | 0.267 ±0.219 | 0.187 ±0.178 | 0.100 ±0.108 | 0.049 ±0.086 | 0.025 ±0.047 | 0.002 ±0.008 | 0.000 ±0.000 |
| 6 | 0.391 ±0.227 | 0.313 ±0.224 | 0.247 ±0.198 | 0.158 ±0.164 | 0.090 ±0.110 | 0.050 ±0.079 | 0.012 ±0.029 | 0.000 ±0.000 | 0.000 ±0.000 |
| 6.2 | 0.320 ±0.225 | 0.251 ±0.206 | 0.206 ±0.198 | 0.125 ±0.133 | 0.070 ±0.104 | 0.038 ±0.063 | 0.010 ±0.024 | 0.000 ±0.000 | 0.000 ±0.000 |
| 6.4 | 0.271 ±0.216 | 0.219 ±0.191 | 0.159 ±0.156 | 0.109 ±0.125 | 0.067 ±0.081 | 0.032 ±0.049 | 0.009 ±0.022 | 0.002 ±0.009 | 0.000 ±0.000 |
| 6.6 | 0.222 ±0.203 | 0.172 ±0.163 | 0.133 ±0.144 | 0.092 ±0.097 | 0.056 ±0.077 | 0.024 ±0.037 | 0.007 ±0.024 | 0.002 ±0.009 | 0.000 ±0.000 |
| 6.8 | 0.177 ±0.168 | 0.141 ±0.160 | 0.109 ±0.125 | 0.069 ±0.089 | 0.042 ±0.069 | 0.024 ±0.047 | 0.005 ±0.018 | 0.000 ±0.000 | 0.000 ±0.000 |
| 7 | 0.157 ±0.162 | 0.138 ±0.157 | 0.093 ±0.121 | 0.061 ±0.074 | 0.036 ±0.061 | 0.020 ±0.053 | 0.009 ±0.025 | 0.005 ±0.018 | 0.000 ±0.000 |
| 7.2 | 0.119 ±0.147 | 0.105 ±0.136 | 0.067 ±0.089 | 0.047 ±0.070 | 0.025 ±0.054 | 0.021 ±0.054 | 0.007 ±0.028 | 0.002 ±0.009 | 0.000 ±0.000 |
| 7.4 | 0.100 ±0.134 | 0.074 ±0.101 | 0.056 ±0.074 | 0.037 ±0.053 | 0.020 ±0.041 | 0.011 ±0.026 | 0.004 ±0.018 | 0.004 ±0.018 | 0.000 ±0.000 |
| 7.6 | 0.079 ±0.115 | 0.053 ±0.074 | 0.039 ±0.061 | 0.033 ±0.058 | 0.015 ±0.033 | 0.011 ±0.031 | 0.006 ±0.020 | 0.000 ±0.000 | 0.000 ±0.000 |
| 7.8 | 0.062 ±0.079 | 0.039 ±0.065 | 0.028 ±0.056 | 0.020 ±0.039 | 0.011 ±0.026 | 0.006 ±0.015 | 0.002 ±0.010 | 0.000 ±0.000 | 0.000 ±0.000 |
| 8 | 0.046 ±0.066 | 0.032 ±0.054 | 0.018 ±0.038 | 0.013 ±0.025 | 0.009 ±0.021 | 0.004 ±0.013 | 0.002 ±0.008 | 0.000 ±0.000 | 0.000 ±0.000 |
| 8.2 | 0.036 ±0.053 | 0.029 ±0.040 | 0.018 ±0.038 | 0.015 ±0.028 | 0.007 ±0.020 | 0.005 ±0.019 | 0.002 ±0.009 | 0.000 ±0.000 | 0.000 ±0.000 |
| 8.4 | 0.033 ±0.049 | 0.028 ±0.046 | 0.017 ±0.038 | 0.013 ±0.028 | 0.004 ±0.012 | 0.004 ±0.012 | 0.000 ±0.000 | 0.000 ±0.000 | 0.000 ±0.000 |
| 8.6 | 0.020 ±0.038 | 0.019 ±0.038 | 0.010 ±0.023 | 0.007 ±0.020 | 0.002 ±0.009 | 0.000 ±0.000 | 0.000 ±0.000 | 0.000 ±0.000 | 0.000 ±0.000 |
| 8.8 | 0.017 ±0.028 | 0.010 ±0.025 | 0.007 ±0.019 | 0.004 ±0.012 | 0.000 ±0.000 | 0.000 ±0.000 | 0.000 ±0.000 | 0.000 ±0.000 | 0.000 ±0.000 |
| 9 | 0.010 ±0.023 | 0.010 ±0.023 | 0.002 ±0.009 | 0.002 ±0.009 | 0.000 ±0.000 | 0.000 ±0.000 | 0.000 ±0.000 | 0.000 ±0.000 | 0.000 ±0.000 |
| 9.2 | 0.011 ±0.027 | 0.010 ±0.023 | 0.004 ±0.012 | 0.000 ±0.000 | 0.000 ±0.000 | 0.000 ±0.000 | 0.000 ±0.000 | 0.000 ±0.000 | 0.000 ±0.000 |
| 9.4 | 0.007 ±0.021 | 0.004 ±0.012 | 0.000 ±0.000 | 0.000 ±0.000 | 0.000 ±0.000 | 0.000 ±0.000 | 0.000 ±0.000 | 0.000 ±0.000 | 0.000 ±0.000 |
| 9.6 | 0.002 ±0.009 | 0.000 ±0.000 | 0.000 ±0.000 | 0.000 ±0.000 | 0.000 ±0.000 | 0.000 ±0.000 | 0.000 ±0.000 | 0.000 ±0.000 | 0.000 ±0.000 |
| 9.8 | 0.004 ±0.012 | 0.000 ±0.000 | 0.000 ±0.000 | 0.000 ±0.000 | 0.000 ±0.000 | 0.000 ±0.000 | 0.000 ±0.000 | 0.000 ±0.000 | 0.000 ±0.000 |
| 10 | 0.000 ±0.000 | 0.000 ±0.000 | 0.000 ±0.000 | 0.000 ±0.000 | 0.000 ±0.000 | 0.000 ±0.000 | 0.000 ±0.000 | 0.000 ±0.000 | 0.000 ±0.000 |

S7 Table: Adapted F1-scores for the focus tests.

Scores in cells correspond to average adapted F1 +/- standard deviation (n=24 images, N=1 experiment) and τ_1_ refers to the intersection over union threshold above which predictions are considered true positives. Analysis corresponds to S6 Fig and rows shaded in grey mark z-layers -1.8 μm to +2.6 μm.
