# Supplementary material for: aiSEGcell: User-friendly deep learning-based segmentation of nuclei in transmitted light images
Source: PLoS Comput Biol. 2024 Aug 23;20(8):e1012361. doi: 10.1371/journal.pcbi.1012361 (PMC11343410; doi:10.1371/journal.pcbi.1012361)
Supplement: S8 Table — Scores in cells correspond to average conventional F1 +/- standard deviation (n = 24 images, N = 1 experiment) and τ1 refers to the intersection over union threshold above which predictions are considered true positives. Analysis corresponds to S6 Fig and rows shaded in grey mark z-layers -1.8 μm to +2.6 μm. (DOCX) [file pcbi.1012361.s024.docx]

| Focal plane [μm] | τ_1_=0.5 | τ_1_=0.55 | τ_1_=0.6 | τ_1_=0.65 | τ_1_=0.7 | τ_1_=0.75 | τ_1_=0.8 | τ_1_=0.85 | τ_1_=0.9 |
| --- | --- | --- | --- | --- | --- | --- | --- | --- | --- |
| -10 | 0.002 ±0.008 | 0.002 ±0.008 | 0.000 ±0.000 | 0.000 ±0.000 | 0.000 ±0.000 | 0.000 ±0.000 | 0.000 ±0.000 | 0.000 ±0.000 | 0.000 ±0.000 |
| -9.8 | 0.000 ±0.000 | 0.000 ±0.000 | 0.000 ±0.000 | 0.000 ±0.000 | 0.000 ±0.000 | 0.000 ±0.000 | 0.000 ±0.000 | 0.000 ±0.000 | 0.000 ±0.000 |
| -9.6 | 0.000 ±0.000 | 0.000 ±0.000 | 0.000 ±0.000 | 0.000 ±0.000 | 0.000 ±0.000 | 0.000 ±0.000 | 0.000 ±0.000 | 0.000 ±0.000 | 0.000 ±0.000 |
| -9.4 | 0.002 ±0.010 | 0.002 ±0.010 | 0.002 ±0.010 | 0.000 ±0.000 | 0.000 ±0.000 | 0.000 ±0.000 | 0.000 ±0.000 | 0.000 ±0.000 | 0.000 ±0.000 |
| -9.2 | 0.000 ±0.000 | 0.000 ±0.000 | 0.000 ±0.000 | 0.000 ±0.000 | 0.000 ±0.000 | 0.000 ±0.000 | 0.000 ±0.000 | 0.000 ±0.000 | 0.000 ±0.000 |
| -9 | 0.000 ±0.000 | 0.000 ±0.000 | 0.000 ±0.000 | 0.000 ±0.000 | 0.000 ±0.000 | 0.000 ±0.000 | 0.000 ±0.000 | 0.000 ±0.000 | 0.000 ±0.000 |
| -8.8 | 0.002 ±0.008 | 0.002 ±0.008 | 0.002 ±0.008 | 0.000 ±0.000 | 0.000 ±0.000 | 0.000 ±0.000 | 0.000 ±0.000 | 0.000 ±0.000 | 0.000 ±0.000 |
| -8.6 | 0.002 ±0.007 | 0.000 ±0.000 | 0.000 ±0.000 | 0.000 ±0.000 | 0.000 ±0.000 | 0.000 ±0.000 | 0.000 ±0.000 | 0.000 ±0.000 | 0.000 ±0.000 |
| -8.4 | 0.007 ±0.016 | 0.005 ±0.015 | 0.003 ±0.011 | 0.002 ±0.009 | 0.000 ±0.000 | 0.000 ±0.000 | 0.000 ±0.000 | 0.000 ±0.000 | 0.000 ±0.000 |
| -8.2 | 0.008 ±0.019 | 0.003 ±0.010 | 0.002 ±0.007 | 0.002 ±0.007 | 0.002 ±0.007 | 0.000 ±0.000 | 0.000 ±0.000 | 0.000 ±0.000 | 0.000 ±0.000 |
| -8 | 0.005 ±0.013 | 0.005 ±0.013 | 0.005 ±0.013 | 0.005 ±0.013 | 0.005 ±0.013 | 0.002 ±0.007 | 0.000 ±0.000 | 0.000 ±0.000 | 0.000 ±0.000 |
| -7.8 | 0.005 ±0.018 | 0.005 ±0.018 | 0.004 ±0.014 | 0.002 ±0.012 | 0.000 ±0.000 | 0.000 ±0.000 | 0.000 ±0.000 | 0.000 ±0.000 | 0.000 ±0.000 |
| -7.6 | 0.011 ±0.024 | 0.011 ±0.024 | 0.003 ±0.010 | 0.003 ±0.010 | 0.001 ±0.007 | 0.001 ±0.007 | 0.001 ±0.007 | 0.001 ±0.007 | 0.000 ±0.000 |
| -7.4 | 0.009 ±0.020 | 0.008 ±0.019 | 0.008 ±0.019 | 0.005 ±0.013 | 0.002 ±0.008 | 0.000 ±0.000 | 0.000 ±0.000 | 0.000 ±0.000 | 0.000 ±0.000 |
| -7.2 | 0.005 ±0.013 | 0.003 ±0.011 | 0.001 ±0.007 | 0.001 ±0.007 | 0.001 ±0.007 | 0.000 ±0.000 | 0.000 ±0.000 | 0.000 ±0.000 | 0.000 ±0.000 |
| -7 | 0.011 ±0.020 | 0.007 ±0.016 | 0.005 ±0.012 | 0.001 ±0.007 | 0.001 ±0.007 | 0.000 ±0.000 | 0.000 ±0.000 | 0.000 ±0.000 | 0.000 ±0.000 |
| -6.8 | 0.013 ±0.026 | 0.010 ±0.025 | 0.008 ±0.018 | 0.003 ±0.011 | 0.002 ±0.009 | 0.000 ±0.000 | 0.000 ±0.000 | 0.000 ±0.000 | 0.000 ±0.000 |
| -6.6 | 0.020 ±0.041 | 0.009 ±0.019 | 0.004 ±0.013 | 0.004 ±0.013 | 0.002 ±0.009 | 0.000 ±0.000 | 0.000 ±0.000 | 0.000 ±0.000 | 0.000 ±0.000 |
| -6.4 | 0.015 ±0.031 | 0.007 ±0.017 | 0.007 ±0.017 | 0.006 ±0.014 | 0.001 ±0.005 | 0.000 ±0.000 | 0.000 ±0.000 | 0.000 ±0.000 | 0.000 ±0.000 |
| -6.2 | 0.030 ±0.030 | 0.021 ±0.026 | 0.007 ±0.016 | 0.003 ±0.011 | 0.002 ±0.010 | 0.002 ±0.010 | 0.000 ±0.000 | 0.000 ±0.000 | 0.000 ±0.000 |
| -6 | 0.021 ±0.034 | 0.014 ±0.024 | 0.009 ±0.016 | 0.008 ±0.016 | 0.003 ±0.011 | 0.000 ±0.000 | 0.000 ±0.000 | 0.000 ±0.000 | 0.000 ±0.000 |
| -5.8 | 0.029 ±0.053 | 0.017 ±0.035 | 0.012 ±0.024 | 0.010 ±0.017 | 0.007 ±0.015 | 0.004 ±0.012 | 0.000 ±0.000 | 0.000 ±0.000 | 0.000 ±0.000 |
| -5.6 | 0.054 ±0.059 | 0.032 ±0.048 | 0.022 ±0.035 | 0.007 ±0.020 | 0.002 ±0.007 | 0.002 ±0.007 | 0.001 ±0.005 | 0.000 ±0.000 | 0.000 ±0.000 |
| -5.4 | 0.057 ±0.071 | 0.038 ±0.052 | 0.024 ±0.036 | 0.014 ±0.026 | 0.007 ±0.019 | 0.002 ±0.008 | 0.000 ±0.000 | 0.000 ±0.000 | 0.000 ±0.000 |
| -5.2 | 0.082 ±0.092 | 0.049 ±0.062 | 0.028 ±0.035 | 0.015 ±0.030 | 0.009 ±0.022 | 0.004 ±0.013 | 0.001 ±0.005 | 0.001 ±0.005 | 0.000 ±0.000 |
| -5 | 0.099 ±0.098 | 0.067 ±0.081 | 0.039 ±0.046 | 0.029 ±0.036 | 0.017 ±0.024 | 0.003 ±0.008 | 0.001 ±0.004 | 0.001 ±0.004 | 0.000 ±0.000 |
| -4.8 | 0.118 ±0.108 | 0.083 ±0.089 | 0.056 ±0.061 | 0.025 ±0.038 | 0.016 ±0.024 | 0.008 ±0.018 | 0.001 ±0.004 | 0.000 ±0.000 | 0.000 ±0.000 |
| -4.6 | 0.155 ±0.129 | 0.120 ±0.106 | 0.078 ±0.078 | 0.042 ±0.051 | 0.021 ±0.036 | 0.011 ±0.025 | 0.006 ±0.015 | 0.002 ±0.007 | 0.000 ±0.000 |
| -4.4 | 0.184 ±0.129 | 0.134 ±0.123 | 0.096 ±0.095 | 0.059 ±0.065 | 0.031 ±0.043 | 0.014 ±0.027 | 0.008 ±0.019 | 0.002 ±0.007 | 0.000 ±0.000 |
| -4.2 | 0.245 ±0.168 | 0.185 ±0.132 | 0.118 ±0.104 | 0.074 ±0.079 | 0.032 ±0.045 | 0.014 ±0.032 | 0.004 ±0.014 | 0.001 ±0.004 | 0.001 ±0.004 |
| -4 | 0.282 ±0.198 | 0.219 ±0.163 | 0.152 ±0.121 | 0.099 ±0.085 | 0.053 ±0.049 | 0.021 ±0.031 | 0.006 ±0.013 | 0.000 ±0.000 | 0.000 ±0.000 |
| -3.8 | 0.322 ±0.187 | 0.250 ±0.161 | 0.189 ±0.132 | 0.130 ±0.106 | 0.067 ±0.064 | 0.040 ±0.042 | 0.016 ±0.022 | 0.005 ±0.013 | 0.000 ±0.000 |
| -3.6 | 0.367 ±0.213 | 0.299 ±0.186 | 0.225 ±0.151 | 0.145 ±0.107 | 0.096 ±0.081 | 0.049 ±0.046 | 0.018 ±0.022 | 0.006 ±0.014 | 0.000 ±0.000 |
| -3.4 | 0.418 ±0.203 | 0.344 ±0.180 | 0.251 ±0.147 | 0.172 ±0.118 | 0.108 ±0.091 | 0.052 ±0.056 | 0.023 ±0.033 | 0.006 ±0.012 | 0.000 ±0.000 |
| -3.2 | 0.499 ±0.178 | 0.405 ±0.172 | 0.315 ±0.152 | 0.218 ±0.134 | 0.137 ±0.105 | 0.072 ±0.070 | 0.032 ±0.038 | 0.007 ±0.013 | 0.002 ±0.006 |
| -3 | 0.558 ±0.177 | 0.467 ±0.176 | 0.369 ±0.163 | 0.262 ±0.142 | 0.164 ±0.112 | 0.103 ±0.096 | 0.044 ±0.050 | 0.011 ±0.030 | 0.000 ±0.000 |
| -2.8 | 0.645 ±0.164 | 0.550 ±0.158 | 0.434 ±0.168 | 0.311 ±0.149 | 0.216 ±0.113 | 0.120 ±0.087 | 0.053 ±0.055 | 0.011 ±0.017 | 0.000 ±0.000 |
| -2.6 | 0.686 ±0.154 | 0.612 ±0.174 | 0.514 ±0.178 | 0.386 ±0.146 | 0.250 ±0.124 | 0.140 ±0.086 | 0.057 ±0.051 | 0.019 ±0.027 | 0.002 ±0.009 |
| -2.4 | 0.741 ±0.134 | 0.676 ±0.147 | 0.563 ±0.175 | 0.434 ±0.153 | 0.310 ±0.133 | 0.177 ±0.099 | 0.082 ±0.063 | 0.028 ±0.027 | 0.004 ±0.008 |
| -2.2 | 0.775 ±0.130 | 0.715 ±0.141 | 0.632 ±0.151 | 0.515 ±0.139 | 0.374 ±0.118 | 0.229 ±0.106 | 0.095 ±0.067 | 0.036 ±0.043 | 0.004 ±0.013 |
| -2 | 0.823 ±0.092 | 0.763 ±0.115 | 0.663 ±0.157 | 0.545 ±0.154 | 0.410 ±0.131 | 0.266 ±0.102 | 0.118 ±0.076 | 0.039 ±0.051 | 0.006 ±0.012 |
| -1.8 | 0.844 ±0.068 | 0.797 ±0.077 | 0.728 ±0.118 | 0.624 ±0.141 | 0.494 ±0.129 | 0.318 ±0.106 | 0.144 ±0.080 | 0.048 ±0.053 | 0.009 ±0.019 |
| -1.6 | 0.864 ±0.064 | 0.830 ±0.065 | 0.756 ±0.080 | 0.657 ±0.118 | 0.533 ±0.129 | 0.354 ±0.112 | 0.180 ±0.087 | 0.068 ±0.049 | 0.011 ±0.017 |
| -1.4 | 0.866 ±0.054 | 0.842 ±0.055 | 0.793 ±0.069 | 0.698 ±0.111 | 0.579 ±0.126 | 0.408 ±0.124 | 0.208 ±0.085 | 0.076 ±0.062 | 0.013 ±0.023 |
| -1.2 | 0.880 ±0.054 | 0.855 ±0.052 | 0.817 ±0.069 | 0.733 ±0.089 | 0.621 ±0.103 | 0.461 ±0.119 | 0.270 ±0.104 | 0.098 ±0.063 | 0.016 ±0.021 |
| -1 | 0.890 ±0.052 | 0.858 ±0.058 | 0.823 ±0.066 | 0.761 ±0.092 | 0.659 ±0.098 | 0.501 ±0.114 | 0.297 ±0.108 | 0.118 ±0.067 | 0.025 ±0.030 |
| -0.8 | 0.894 ±0.048 | 0.873 ±0.050 | 0.846 ±0.055 | 0.782 ±0.081 | 0.667 ±0.096 | 0.523 ±0.093 | 0.333 ±0.096 | 0.140 ±0.070 | 0.023 ±0.029 |
| -0.6 | 0.895 ±0.047 | 0.875 ±0.048 | 0.842 ±0.065 | 0.777 ±0.094 | 0.681 ±0.096 | 0.538 ±0.113 | 0.364 ±0.118 | 0.164 ±0.095 | 0.029 ±0.032 |
| -0.4 | 0.896 ±0.048 | 0.880 ±0.046 | 0.849 ±0.058 | 0.793 ±0.080 | 0.712 ±0.089 | 0.573 ±0.101 | 0.400 ±0.108 | 0.180 ±0.093 | 0.042 ±0.048 |
| -0.2 | 0.894 ±0.046 | 0.886 ±0.048 | 0.846 ±0.059 | 0.785 ±0.069 | 0.703 ±0.094 | 0.573 ±0.083 | 0.385 ±0.104 | 0.181 ±0.094 | 0.031 ±0.033 |
| 0 | 0.891 ±0.046 | 0.882 ±0.048 | 0.852 ±0.054 | 0.802 ±0.057 | 0.717 ±0.075 | 0.596 ±0.080 | 0.420 ±0.088 | 0.198 ±0.081 | 0.044 ±0.043 |
| 0.2 | 0.892 ±0.040 | 0.877 ±0.043 | 0.852 ±0.054 | 0.795 ±0.057 | 0.713 ±0.076 | 0.603 ±0.075 | 0.407 ±0.096 | 0.198 ±0.080 | 0.035 ±0.029 |
| 0.4 | 0.883 ±0.039 | 0.876 ±0.040 | 0.843 ±0.045 | 0.795 ±0.059 | 0.709 ±0.073 | 0.587 ±0.082 | 0.393 ±0.095 | 0.185 ±0.075 | 0.036 ±0.035 |
| 0.6 | 0.889 ±0.042 | 0.878 ±0.043 | 0.853 ±0.052 | 0.787 ±0.074 | 0.693 ±0.083 | 0.588 ±0.072 | 0.396 ±0.092 | 0.183 ±0.097 | 0.024 ±0.031 |
| 0.8 | 0.892 ±0.043 | 0.877 ±0.042 | 0.848 ±0.051 | 0.788 ±0.069 | 0.705 ±0.074 | 0.567 ±0.074 | 0.385 ±0.080 | 0.176 ±0.072 | 0.032 ±0.028 |
| 1 | 0.891 ±0.036 | 0.880 ±0.039 | 0.853 ±0.044 | 0.789 ±0.075 | 0.690 ±0.076 | 0.543 ±0.072 | 0.363 ±0.082 | 0.163 ±0.081 | 0.028 ±0.036 |
| 1.2 | 0.885 ±0.045 | 0.867 ±0.050 | 0.833 ±0.055 | 0.763 ±0.073 | 0.647 ±0.072 | 0.505 ±0.071 | 0.322 ±0.087 | 0.152 ±0.076 | 0.031 ±0.028 |
| 1.4 | 0.892 ±0.044 | 0.873 ±0.052 | 0.830 ±0.056 | 0.763 ±0.068 | 0.647 ±0.068 | 0.485 ±0.093 | 0.293 ±0.089 | 0.126 ±0.066 | 0.018 ±0.020 |
| 1.6 | 0.875 ±0.051 | 0.856 ±0.052 | 0.814 ±0.066 | 0.720 ±0.084 | 0.612 ±0.099 | 0.452 ±0.091 | 0.278 ±0.090 | 0.108 ±0.071 | 0.015 ±0.027 |
| 1.8 | 0.874 ±0.041 | 0.849 ±0.047 | 0.795 ±0.061 | 0.716 ±0.096 | 0.573 ±0.101 | 0.445 ±0.104 | 0.258 ±0.100 | 0.080 ±0.049 | 0.013 ±0.017 |
| 2 | 0.870 ±0.044 | 0.837 ±0.058 | 0.774 ±0.074 | 0.689 ±0.093 | 0.564 ±0.098 | 0.427 ±0.121 | 0.224 ±0.106 | 0.082 ±0.062 | 0.012 ±0.017 |
| 2.2 | 0.865 ±0.051 | 0.830 ±0.063 | 0.758 ±0.082 | 0.674 ±0.097 | 0.541 ±0.115 | 0.372 ±0.114 | 0.205 ±0.087 | 0.060 ±0.047 | 0.008 ±0.016 |
| 2.4 | 0.850 ±0.057 | 0.811 ±0.065 | 0.741 ±0.080 | 0.646 ±0.094 | 0.505 ±0.096 | 0.357 ±0.103 | 0.190 ±0.080 | 0.059 ±0.041 | 0.006 ±0.018 |
| 2.6 | 0.853 ±0.071 | 0.814 ±0.071 | 0.742 ±0.091 | 0.646 ±0.100 | 0.504 ±0.111 | 0.335 ±0.115 | 0.164 ±0.085 | 0.049 ±0.040 | 0.008 ±0.015 |
| 2.8 | 0.833 ±0.074 | 0.780 ±0.082 | 0.698 ±0.096 | 0.609 ±0.111 | 0.457 ±0.103 | 0.294 ±0.103 | 0.146 ±0.070 | 0.052 ±0.045 | 0.007 ±0.014 |
| 3 | 0.823 ±0.061 | 0.775 ±0.080 | 0.706 ±0.094 | 0.577 ±0.113 | 0.454 ±0.116 | 0.267 ±0.098 | 0.142 ±0.079 | 0.046 ±0.038 | 0.004 ±0.011 |
| 3.2 | 0.823 ±0.074 | 0.773 ±0.080 | 0.692 ±0.110 | 0.573 ±0.130 | 0.398 ±0.113 | 0.255 ±0.088 | 0.121 ±0.064 | 0.037 ±0.041 | 0.004 ±0.010 |
| 3.4 | 0.814 ±0.080 | 0.756 ±0.094 | 0.670 ±0.124 | 0.544 ±0.127 | 0.382 ±0.119 | 0.228 ±0.087 | 0.107 ±0.055 | 0.033 ±0.034 | 0.004 ±0.012 |
| 3.6 | 0.789 ±0.087 | 0.737 ±0.096 | 0.652 ±0.120 | 0.514 ±0.135 | 0.344 ±0.108 | 0.198 ±0.078 | 0.085 ±0.062 | 0.024 ±0.029 | 0.001 ±0.004 |
| 3.8 | 0.787 ±0.091 | 0.725 ±0.104 | 0.622 ±0.124 | 0.485 ±0.144 | 0.324 ±0.125 | 0.172 ±0.086 | 0.063 ±0.042 | 0.019 ±0.026 | 0.001 ±0.006 |
| 4 | 0.775 ±0.109 | 0.702 ±0.121 | 0.607 ±0.146 | 0.454 ±0.140 | 0.288 ±0.112 | 0.148 ±0.070 | 0.050 ±0.039 | 0.013 ±0.022 | 0.000 ±0.000 |
| 4.2 | 0.742 ±0.111 | 0.667 ±0.137 | 0.556 ±0.154 | 0.414 ±0.143 | 0.264 ±0.114 | 0.128 ±0.070 | 0.046 ±0.039 | 0.016 ±0.020 | 0.001 ±0.005 |
| 4.4 | 0.717 ±0.127 | 0.636 ±0.141 | 0.536 ±0.144 | 0.390 ±0.144 | 0.232 ±0.104 | 0.104 ±0.061 | 0.038 ±0.036 | 0.007 ±0.019 | 0.001 ±0.004 |
| 4.6 | 0.676 ±0.150 | 0.584 ±0.168 | 0.487 ±0.159 | 0.344 ±0.141 | 0.213 ±0.105 | 0.087 ±0.067 | 0.042 ±0.045 | 0.010 ±0.020 | 0.000 ±0.000 |
| 4.8 | 0.647 ±0.176 | 0.549 ±0.183 | 0.430 ±0.167 | 0.296 ±0.146 | 0.174 ±0.091 | 0.077 ±0.068 | 0.029 ±0.034 | 0.004 ±0.011 | 0.000 ±0.000 |
| 5 | 0.607 ±0.203 | 0.532 ±0.199 | 0.405 ±0.156 | 0.263 ±0.130 | 0.141 ±0.097 | 0.078 ±0.079 | 0.029 ±0.035 | 0.009 ±0.015 | 0.000 ±0.000 |
| 5.2 | 0.538 ±0.209 | 0.458 ±0.193 | 0.338 ±0.163 | 0.233 ±0.139 | 0.112 ±0.097 | 0.052 ±0.060 | 0.022 ±0.033 | 0.008 ±0.018 | 0.000 ±0.000 |
| 5.4 | 0.476 ±0.225 | 0.387 ±0.206 | 0.283 ±0.174 | 0.172 ±0.125 | 0.104 ±0.089 | 0.054 ±0.063 | 0.019 ±0.032 | 0.005 ±0.011 | 0.000 ±0.000 |
| 5.6 | 0.428 ±0.207 | 0.334 ±0.198 | 0.235 ±0.158 | 0.154 ±0.132 | 0.074 ±0.074 | 0.039 ±0.050 | 0.015 ±0.025 | 0.004 ±0.013 | 0.000 ±0.000 |
| 5.8 | 0.382 ±0.239 | 0.273 ±0.199 | 0.209 ±0.184 | 0.136 ±0.135 | 0.067 ±0.072 | 0.032 ±0.055 | 0.016 ±0.029 | 0.001 ±0.005 | 0.000 ±0.000 |
| 6 | 0.329 ±0.199 | 0.251 ±0.191 | 0.188 ±0.156 | 0.112 ±0.117 | 0.060 ±0.072 | 0.032 ±0.050 | 0.007 ±0.018 | 0.000 ±0.000 | 0.000 ±0.000 |
| 6.2 | 0.271 ±0.197 | 0.201 ±0.171 | 0.161 ±0.161 | 0.089 ±0.094 | 0.048 ±0.071 | 0.024 ±0.040 | 0.006 ±0.015 | 0.000 ±0.000 | 0.000 ±0.000 |
| 6.4 | 0.229 ±0.187 | 0.176 ±0.155 | 0.121 ±0.119 | 0.080 ±0.090 | 0.046 ±0.056 | 0.021 ±0.032 | 0.006 ±0.014 | 0.001 ±0.006 | 0.000 ±0.000 |
| 6.6 | 0.190 ±0.177 | 0.139 ±0.131 | 0.104 ±0.112 | 0.069 ±0.073 | 0.041 ±0.057 | 0.017 ±0.027 | 0.006 ±0.019 | 0.001 ±0.007 | 0.000 ±0.000 |
| 6.8 | 0.150 ±0.143 | 0.116 ±0.132 | 0.086 ±0.097 | 0.053 ±0.068 | 0.031 ±0.053 | 0.018 ±0.034 | 0.003 ±0.012 | 0.000 ±0.000 | 0.000 ±0.000 |
| 7 | 0.137 ±0.141 | 0.119 ±0.137 | 0.078 ±0.100 | 0.049 ±0.059 | 0.029 ±0.051 | 0.016 ±0.046 | 0.007 ±0.019 | 0.004 ±0.013 | 0.000 ±0.000 |
| 7.2 | 0.105 ±0.130 | 0.091 ±0.118 | 0.055 ±0.071 | 0.038 ±0.058 | 0.021 ±0.048 | 0.018 ±0.048 | 0.007 ±0.024 | 0.002 ±0.008 | 0.000 ±0.000 |
| 7.4 | 0.090 ±0.120 | 0.065 ±0.087 | 0.047 ±0.062 | 0.031 ±0.046 | 0.017 ±0.036 | 0.009 ±0.022 | 0.003 ±0.015 | 0.003 ±0.015 | 0.000 ±0.000 |
| 7.6 | 0.071 ±0.105 | 0.046 ±0.064 | 0.033 ±0.053 | 0.029 ±0.049 | 0.013 ±0.029 | 0.009 ±0.027 | 0.005 ±0.018 | 0.000 ±0.000 | 0.000 ±0.000 |
| 7.8 | 0.055 ±0.069 | 0.035 ±0.057 | 0.024 ±0.047 | 0.018 ±0.034 | 0.009 ±0.022 | 0.005 ±0.014 | 0.002 ±0.010 | 0.000 ±0.000 | 0.000 ±0.000 |
| 8 | 0.043 ±0.061 | 0.029 ±0.049 | 0.017 ±0.033 | 0.012 ±0.022 | 0.008 ±0.018 | 0.004 ±0.013 | 0.002 ±0.008 | 0.000 ±0.000 | 0.000 ±0.000 |
| 8.2 | 0.034 ±0.049 | 0.027 ±0.037 | 0.017 ±0.035 | 0.014 ±0.027 | 0.007 ±0.020 | 0.005 ±0.019 | 0.002 ±0.009 | 0.000 ±0.000 | 0.000 ±0.000 |
| 8.4 | 0.031 ±0.047 | 0.027 ±0.043 | 0.015 ±0.035 | 0.012 ±0.026 | 0.003 ±0.012 | 0.003 ±0.012 | 0.000 ±0.000 | 0.000 ±0.000 | 0.000 ±0.000 |
| 8.6 | 0.020 ±0.037 | 0.018 ±0.037 | 0.009 ±0.022 | 0.007 ±0.019 | 0.002 ±0.009 | 0.000 ±0.000 | 0.000 ±0.000 | 0.000 ±0.000 | 0.000 ±0.000 |
| 8.8 | 0.016 ±0.027 | 0.010 ±0.025 | 0.007 ±0.019 | 0.004 ±0.012 | 0.000 ±0.000 | 0.000 ±0.000 | 0.000 ±0.000 | 0.000 ±0.000 | 0.000 ±0.000 |
| 9 | 0.010 ±0.023 | 0.010 ±0.023 | 0.002 ±0.009 | 0.002 ±0.009 | 0.000 ±0.000 | 0.000 ±0.000 | 0.000 ±0.000 | 0.000 ±0.000 | 0.000 ±0.000 |
| 9.2 | 0.011 ±0.027 | 0.010 ±0.023 | 0.004 ±0.012 | 0.000 ±0.000 | 0.000 ±0.000 | 0.000 ±0.000 | 0.000 ±0.000 | 0.000 ±0.000 | 0.000 ±0.000 |
| 9.4 | 0.007 ±0.021 | 0.004 ±0.012 | 0.000 ±0.000 | 0.000 ±0.000 | 0.000 ±0.000 | 0.000 ±0.000 | 0.000 ±0.000 | 0.000 ±0.000 | 0.000 ±0.000 |
| 9.6 | 0.002 ±0.009 | 0.000 ±0.000 | 0.000 ±0.000 | 0.000 ±0.000 | 0.000 ±0.000 | 0.000 ±0.000 | 0.000 ±0.000 | 0.000 ±0.000 | 0.000 ±0.000 |
| 9.8 | 0.004 ±0.012 | 0.000 ±0.000 | 0.000 ±0.000 | 0.000 ±0.000 | 0.000 ±0.000 | 0.000 ±0.000 | 0.000 ±0.000 | 0.000 ±0.000 | 0.000 ±0.000 |
| 10 | 0.000 ±0.000 | 0.000 ±0.000 | 0.000 ±0.000 | 0.000 ±0.000 | 0.000 ±0.000 | 0.000 ±0.000 | 0.000 ±0.000 | 0.000 ±0.000 | 0.000 ±0.000 |

S8 Table: Conventional F1-scores for the focus tests.

Scores in cells correspond to average conventional F1 +/- standard deviation (n=24 images, N=1 experiment) and τ_1_ refers to the intersection over union threshold above which predictions are considered true positives. Analysis corresponds to S6 Fig and rows shaded in grey mark z-layers -1.8 μm to +2.6 μm.
