# Supplementary material for: aiSEGcell: User-friendly deep learning-based segmentation of nuclei in transmitted light images
Source: PLoS Comput Biol. 2024 Aug 23;20(8):e1012361. doi: 10.1371/journal.pcbi.1012361 (PMC11343410; doi:10.1371/journal.pcbi.1012361)
Supplement: S10 Table — Scores in cells correspond to average conventional F1 +/- standard deviation (n = 10 images, N = 1 experiment) and τ1 refers to the intersection over union threshold above which predictions are considered true positives (best model per τ1 in bold). The green shaded row corresponds to the model we selected for testing and the green square in S7a Fig. (DOCX) [file pcbi.1012361.s026.docx]

| Loss weight | τ_1_=0.5 | τ_1_=0.55 | τ_1_=0.6 | τ_1_=0.65 | τ_1_=0.7 | τ_1_=0.75 | τ_1_=0.8 | τ_1_=0.85 | τ_1_=0.9 |
| --- | --- | --- | --- | --- | --- | --- | --- | --- | --- |
| 1 | 0.865 ±0.053 | 0.847 ±0.063 | 0.820 ±0.071 | 0.763 ±0.093 | 0.665 ±0.109 | 0.534 ±0.107 | 0.347 ±0.096 | 0.163 ±0.046 | 0.028 ±0.013 |
| 2 | 0.847 ±0.056 | 0.821 ±0.068 | 0.783 ±0.078 | 0.716 ±0.085 | 0.610 ±0.107 | 0.470 ±0.116 | 0.289 ±0.106 | 0.134 ±0.052 | 0.030 ±0.018 |
| 3 | 0.835 ±0.060 | 0.794 ±0.064 | 0.726 ±0.088 | 0.620 ±0.106 | 0.503 ±0.094 | 0.333 ±0.079 | 0.179 ±0.048 | 0.081 ±0.038 | 0.026 ±0.020 |
| 4 | 0.832 ±0.069 | 0.783 ±0.078 | 0.726 ±0.089 | 0.636 ±0.106 | 0.507 ±0.108 | 0.348 ±0.094 | 0.194 ±0.056 | 0.076 ±0.031 | 0.020 ±0.015 |
| 5 | 0.815 ±0.060 | 0.773 ±0.083 | 0.708 ±0.085 | 0.612 ±0.103 | 0.459 ±0.093 | 0.304 ±0.089 | 0.165 ±0.068 | 0.083 ±0.039 | 0.021 ±0.021 |
| 6 | 0.845 ±0.056 | 0.794 ±0.076 | 0.719 ±0.090 | 0.618 ±0.107 | 0.508 ±0.103 | 0.356 ±0.080 | 0.215 ±0.059 | 0.115 ±0.037 | 0.027 ±0.013 |
| 7 | 0.832 ±0.056 | 0.780 ±0.066 | 0.688 ±0.095 | 0.573 ±0.103 | 0.440 ±0.105 | 0.292 ±0.082 | 0.159 ±0.060 | 0.065 ±0.028 | 0.023 ±0.015 |
| 8 | 0.808 ±0.059 | 0.769 ±0.074 | 0.689 ±0.084 | 0.607 ±0.094 | 0.499 ±0.083 | 0.347 ±0.073 | 0.208 ±0.045 | 0.098 ±0.028 | 0.027 ±0.016 |
| 9 | 0.817 ±0.070 | 0.758 ±0.087 | 0.680 ±0.079 | 0.567 ±0.086 | 0.426 ±0.074 | 0.279 ±0.075 | 0.159 ±0.050 | 0.073 ±0.033 | 0.027 ±0.018 |
| 10 | 0.798 ±0.072 | 0.743 ±0.080 | 0.671 ±0.091 | 0.570 ±0.098 | 0.427 ±0.079 | 0.288 ±0.067 | 0.168 ±0.060 | 0.076 ±0.040 | 0.021 ±0.015 |
| 20 | 0.775 ±0.069 | 0.684 ±0.079 | 0.558 ±0.083 | 0.407 ±0.092 | 0.264 ±0.076 | 0.139 ±0.055 | 0.068 ±0.034 | 0.033 ±0.021 | 0.014 ±0.016 |
| 50 | 0.699 ±0.092 | 0.596 ±0.089 | 0.461 ±0.089 | 0.328 ±0.067 | 0.220 ±0.070 | 0.131 ±0.053 | 0.072 ±0.043 | 0.035 ±0.023 | 0.011 ±0.014 |
| 100 | 0.623 ±0.097 | 0.492 ±0.095 | 0.340 ±0.087 | 0.208 ±0.065 | 0.110 ±0.040 | 0.060 ±0.030 | 0.040 ±0.020 | 0.025 ±0.016 | 0.012 ±0.012 |
| 150 | 0.604 ±0.092 | 0.464 ±0.082 | 0.331 ±0.075 | 0.201 ±0.059 | 0.117 ±0.049 | 0.071 ±0.033 | 0.038 ±0.017 | 0.021 ±0.015 | 0.007 ±0.007 |
| 200 | 0.550 ±0.084 | 0.408 ±0.082 | 0.275 ±0.070 | 0.164 ±0.051 | 0.098 ±0.042 | 0.059 ±0.031 | 0.034 ±0.019 | 0.019 ±0.014 | 0.009 ±0.010 |
| 1 | 0.865 ±0.065 | 0.845 ±0.073 | 0.807 ±0.090 | 0.743 ±0.108 | 0.641 ±0.118 | 0.498 ±0.102 | 0.316 ±0.082 | 0.146 ±0.047 | 0.030 ±0.018 |
| 2 | 0.858 ±0.057 | 0.822 ±0.069 | 0.780 ±0.088 | 0.710 ±0.098 | 0.597 ±0.107 | 0.434 ±0.081 | 0.247 ±0.067 | 0.107 ±0.034 | 0.032 ±0.020 |
| 3 | 0.844 ±0.064 | 0.808 ±0.079 | 0.754 ±0.095 | 0.667 ±0.114 | 0.560 ±0.123 | 0.418 ±0.093 | 0.245 ±0.078 | 0.114 ±0.038 | 0.027 ±0.014 |
| 4 | 0.834 ±0.067 | 0.801 ±0.084 | 0.743 ±0.101 | 0.668 ±0.104 | 0.533 ±0.095 | 0.362 ±0.083 | 0.224 ±0.059 | 0.097 ±0.033 | 0.032 ±0.028 |
| 5 | 0.841 ±0.053 | 0.807 ±0.066 | 0.757 ±0.082 | 0.677 ±0.098 | 0.566 ±0.121 | 0.409 ±0.100 | 0.249 ±0.074 | 0.115 ±0.052 | 0.021 ±0.013 |
| 6 | 0.849 ±0.055 | 0.805 ±0.083 | 0.755 ±0.089 | 0.655 ±0.100 | 0.533 ±0.096 | 0.370 ±0.080 | 0.217 ±0.049 | 0.088 ±0.025 | 0.025 ±0.018 |
| 7 | 0.832 ±0.066 | 0.785 ±0.079 | 0.709 ±0.089 | 0.614 ±0.087 | 0.472 ±0.077 | 0.336 ±0.087 | 0.180 ±0.069 | 0.084 ±0.039 | 0.022 ±0.015 |
| 8 | 0.809 ±0.066 | 0.759 ±0.071 | 0.672 ±0.073 | 0.550 ±0.091 | 0.397 ±0.078 | 0.255 ±0.050 | 0.135 ±0.053 | 0.064 ±0.036 | 0.017 ±0.020 |
| 9 | 0.811 ±0.062 | 0.744 ±0.082 | 0.658 ±0.094 | 0.522 ±0.112 | 0.395 ±0.092 | 0.242 ±0.079 | 0.131 ±0.057 | 0.061 ±0.030 | 0.018 ±0.017 |
| 10 | 0.802 ±0.066 | 0.735 ±0.088 | 0.658 ±0.093 | 0.540 ±0.076 | 0.398 ±0.068 | 0.251 ±0.062 | 0.143 ±0.067 | 0.069 ±0.045 | 0.021 ±0.014 |
| 20 | 0.741 ±0.081 | 0.652 ±0.099 | 0.528 ±0.097 | 0.399 ±0.070 | 0.275 ±0.046 | 0.159 ±0.037 | 0.080 ±0.028 | 0.038 ±0.021 | 0.018 ±0.015 |
| 50 | 0.736 ±0.080 | 0.620 ±0.077 | 0.495 ±0.066 | 0.347 ±0.054 | 0.226 ±0.059 | 0.127 ±0.054 | 0.063 ±0.029 | 0.033 ±0.018 | 0.014 ±0.013 |
| 100 | 0.603 ±0.082 | 0.487 ±0.065 | 0.347 ±0.078 | 0.231 ±0.063 | 0.135 ±0.057 | 0.071 ±0.034 | 0.044 ±0.023 | 0.022 ±0.016 | 0.009 ±0.007 |
| 150 | 0.632 ±0.096 | 0.501 ±0.080 | 0.368 ±0.069 | 0.251 ±0.055 | 0.163 ±0.055 | 0.088 ±0.037 | 0.050 ±0.018 | 0.020 ±0.013 | 0.007 ±0.007 |
| 200 | 0.501 ±0.083 | 0.376 ±0.074 | 0.244 ±0.061 | 0.156 ±0.056 | 0.100 ±0.039 | 0.055 ±0.024 | 0.034 ±0.019 | 0.017 ±0.011 | 0.006 ±0.007 |
| 1 | **0.867 ±0.063** | **0.852 ±0.068** | **0.825 ±0.077** | **0.790 ±0.090** | **0.716 ±0.094** | **0.603 ±0.100** | **0.426 ±0.097** | **0.208 ±0.079** | **0.050 ±0.025** |
| 2 | 0.862 ±0.062 | 0.838 ±0.072 | 0.800 ±0.088 | 0.737 ±0.091 | 0.639 ±0.099 | 0.499 ±0.105 | 0.314 ±0.072 | 0.145 ±0.052 | 0.035 ±0.019 |
| 3 | 0.851 ±0.058 | 0.818 ±0.066 | 0.760 ±0.081 | 0.672 ±0.101 | 0.537 ±0.110 | 0.378 ±0.087 | 0.221 ±0.080 | 0.090 ±0.046 | 0.022 ±0.017 |
| 4 | 0.820 ±0.072 | 0.782 ±0.083 | 0.717 ±0.094 | 0.626 ±0.105 | 0.498 ±0.106 | 0.346 ±0.107 | 0.213 ±0.079 | 0.104 ±0.053 | 0.026 ±0.015 |
| 5 | 0.829 ±0.059 | 0.795 ±0.070 | 0.743 ±0.082 | 0.652 ±0.088 | 0.525 ±0.089 | 0.372 ±0.079 | 0.219 ±0.062 | 0.104 ±0.041 | 0.031 ±0.019 |
| 6 | 0.816 ±0.066 | 0.778 ±0.078 | 0.703 ±0.095 | 0.604 ±0.099 | 0.480 ±0.091 | 0.334 ±0.076 | 0.190 ±0.065 | 0.087 ±0.046 | 0.022 ±0.014 |
| 7 | 0.759 ±0.053 | 0.692 ±0.065 | 0.591 ±0.078 | 0.488 ±0.070 | 0.347 ±0.058 | 0.226 ±0.054 | 0.123 ±0.047 | 0.053 ±0.029 | 0.016 ±0.012 |
| 8 | 0.791 ±0.060 | 0.731 ±0.080 | 0.648 ±0.086 | 0.536 ±0.092 | 0.382 ±0.083 | 0.230 ±0.063 | 0.125 ±0.044 | 0.063 ±0.030 | 0.018 ±0.015 |
| 9 | 0.824 ±0.076 | 0.778 ±0.093 | 0.700 ±0.098 | 0.579 ±0.109 | 0.428 ±0.097 | 0.268 ±0.073 | 0.137 ±0.048 | 0.065 ±0.036 | 0.021 ±0.018 |
| 10 | 0.814 ±0.050 | 0.770 ±0.072 | 0.683 ±0.077 | 0.568 ±0.109 | 0.441 ±0.097 | 0.265 ±0.070 | 0.148 ±0.047 | 0.060 ±0.015 | 0.018 ±0.015 |
| 20 | 0.769 ±0.069 | 0.691 ±0.091 | 0.572 ±0.099 | 0.435 ±0.080 | 0.295 ±0.066 | 0.183 ±0.057 | 0.091 ±0.043 | 0.041 ±0.021 | 0.018 ±0.014 |
| 50 | 0.685 ±0.079 | 0.561 ±0.088 | 0.439 ±0.076 | 0.293 ±0.063 | 0.170 ±0.051 | 0.096 ±0.043 | 0.048 ±0.024 | 0.021 ±0.014 | 0.007 ±0.009 |
| 100 | 0.650 ±0.089 | 0.534 ±0.083 | 0.393 ±0.072 | 0.254 ±0.052 | 0.154 ±0.046 | 0.086 ±0.038 | 0.043 ±0.024 | 0.020 ±0.013 | 0.008 ±0.008 |
| 150 | 0.610 ±0.090 | 0.479 ±0.078 | 0.352 ±0.066 | 0.238 ±0.069 | 0.133 ±0.054 | 0.080 ±0.036 | 0.047 ±0.019 | 0.020 ±0.012 | 0.008 ±0.007 |
| 200 | 0.576 ±0.086 | 0.433 ±0.080 | 0.280 ±0.068 | 0.159 ±0.060 | 0.090 ±0.042 | 0.054 ±0.035 | 0.033 ±0.019 | 0.019 ±0.014 | 0.008 ±0.009 |

S10 Table: Conventional F1-scores for the D3 validation set.

Scores in cells correspond to average conventional F1 +/- standard deviation (n=10 images, N=1 experiment) and τ_1_ refers to the intersection over union threshold above which predictions are considered true positives (best model per τ_1_ in bold). The green shaded row corresponds to the model we selected for testing and the green square in S7a Fig.
