# Supplementary material for: aiSEGcell: User-friendly deep learning-based segmentation of nuclei in transmitted light images
Source: PLoS Comput Biol. 2024 Aug 23;20(8):e1012361. doi: 10.1371/journal.pcbi.1012361 (PMC11343410; doi:10.1371/journal.pcbi.1012361)
Supplement: S11 Table — Scores in cells correspond to average conventional or adapted F1 +/- standard deviation (n = 29 images, N = 1 experiment) and τ1 refers to the intersection over union threshold above which predictions are considered true positives. Rows of best models trained on D1 (cyan) and retrained on D3 (green) are shaded and correspond to the respectively colored squares in S1 and S7a Figs. (DOCX) [file pcbi.1012361.s027.docx]

| Model | Score | τ_1_=0.5 | τ_1_=0.55 | τ_1_=0.6 | τ_1_=0.65 | τ_1_=0.7 | τ_1_=0.75 | τ_1_=0.8 | τ_1_=0.85 | τ_1_=0.9 |
| --- | --- | --- | --- | --- | --- | --- | --- | --- | --- | --- |
| D1 trained | adapted | 0.340 ±0.069 | 0.293 ±0.073 | 0.221 ±0.076 | 0.157 ±0.068 | 0.100 ±0.068 | 0.062 ±0.053 | 0.023 ±0.027 | 0.005 ±0.012 | 0.000 ±0.000 |
| D3 re-trained | adapted | 0.741 ±0.062 | 0.724 ±0.064 | 0.694 ±0.068 | 0.640 ±0.071 | 0.567 ±0.070 | 0.424 ±0.076 | 0.288 ±0.087 | 0.143 ±0.062 | 0.039 ±0.039 |
| D1 trained | conventional | 0.295 ±0.068 | 0.246 ±0.066 | 0.179 ±0.065 | 0.122 ±0.056 | 0.076 ±0.054 | 0.047 ±0.040 | 0.017 ±0.020 | 0.003 ±0.009 | 0.000 ±0.000 |
| D3 re-trained | conventional | 0.666 ±0.075 | 0.641 ±0.072 | 0.600 ±0.070 | 0.532 ±0.076 | 0.448 ±0.072 | 0.306 ±0.066 | 0.192 ±0.062 | 0.088 ±0.042 | 0.023 ±0.023 |

S11 Table: F1-scores for the D3 test set.

Scores in cells correspond to average conventional or adapted F1 +/- standard deviation (n=29 images, N=1 experiment) and τ_1_ refers to the intersection over union threshold above which predictions are considered true positives. Rows of best models trained on D1 (cyan) and retrained on D3 (green) are shaded and correspond to the respectively colored squares in Figs S1 and S7a.
