# Supplementary material for: aiSEGcell: User-friendly deep learning-based segmentation of nuclei in transmitted light images
Source: PLoS Comput Biol. 2024 Aug 23;20(8):e1012361. doi: 10.1371/journal.pcbi.1012361 (PMC11343410; doi:10.1371/journal.pcbi.1012361)
Supplement: S13 Table — Scores in cells correspond to average conventional F1 +/- standard deviation (n = 10 images, N = 1 experiment) and τ1 refers to the intersection over union threshold above which predictions are considered true positives (best model per τ1 in bold). The purple shaded row corresponds to the model we selected for testing and the purple square in S7b Fig. List of abbreviations: learning rate (Lr). (DOCX) [file pcbi.1012361.s029.docx]

| Loss weight | Lr | τ_1_=0.5 | τ_1_=0.55 | τ_1_=0.6 | τ_1_=0.65 | τ_1_=0.7 | τ_1_=0.75 | τ_1_=0.8 | τ_1_=0.85 | τ_1_=0.9 |
| --- | --- | --- | --- | --- | --- | --- | --- | --- | --- | --- |
| 1 | 5.00E-05 | 0.932 ±0.020 | 0.915 ±0.025 | 0.894 ±0.024 | 0.852 ±0.038 | 0.781 ±0.038 | 0.646 ±0.063 | 0.489 ±0.066 | 0.290 ±0.054 | 0.080 ±0.019 |
| 2 | 5.00E-05 | 0.932 ±0.030 | 0.919 ±0.029 | 0.903 ±0.027 | 0.860 ±0.035 | 0.799 ±0.043 | 0.680 ±0.068 | 0.515 ±0.069 | 0.307 ±0.055 | 0.090 ±0.024 |
| 3 | 5.00E-05 | 0.925 ±0.018 | 0.917 ±0.015 | 0.899 ±0.014 | 0.870 ±0.024 | **0.830 ±0.022** | **0.740 ±0.031** | **0.573 ±0.060** | 0.345 ±0.058 | 0.109 ±0.029 |
| 1 | 1.00E-05 | 0.937 ±0.024 | 0.920 ±0.020 | 0.899 ±0.020 | 0.847 ±0.043 | 0.775 ±0.048 | 0.657 ±0.069 | 0.478 ±0.059 | 0.279 ±0.047 | 0.076 ±0.024 |
| 2 | 1.00E-05 | 0.929 ±0.026 | 0.918 ±0.029 | 0.901 ±0.024 | 0.864 ±0.025 | 0.818 ±0.040 | 0.716 ±0.053 | 0.560 ±0.053 | **0.348 ±0.068** | 0.106 ±0.045 |
| 3 | 1.00E-05 | 0.924 ±0.022 | 0.909 ±0.023 | 0.890 ±0.019 | 0.854 ±0.037 | 0.810 ±0.038 | 0.709 ±0.038 | 0.537 ±0.045 | 0.283 ±0.051 | 0.084 ±0.037 |
| 1 | 5.00E-06 | 0.931 ±0.018 | 0.918 ±0.022 | 0.887 ±0.024 | 0.838 ±0.043 | 0.772 ±0.052 | 0.663 ±0.069 | 0.501 ±0.073 | 0.284 ±0.053 | 0.077 ±0.052 |
| 2 | 5.00E-06 | 0.928 ±0.023 | 0.915 ±0.022 | 0.893 ±0.020 | 0.866 ±0.032 | 0.815 ±0.037 | 0.712 ±0.061 | 0.551 ±0.048 | 0.318 ±0.048 | 0.103 ±0.031 |
| 3 | 5.00E-06 | 0.929 ±0.022 | 0.920 ±0.020 | 0.901 ±0.023 | 0.863 ±0.031 | 0.798 ±0.046 | 0.710 ±0.047 | 0.528 ±0.046 | 0.304 ±0.051 | 0.105 ±0.026 |
| 1 | 1.00E-06 | 0.930 ±0.024 | 0.913 ±0.018 | 0.892 ±0.020 | 0.853 ±0.034 | 0.785 ±0.035 | 0.673 ±0.058 | 0.516 ±0.077 | 0.311 ±0.048 | 0.110 ±0.028 |
| 2 | 1.00E-06 | 0.929 ±0.033 | 0.915 ±0.029 | 0.892 ±0.024 | 0.858 ±0.034 | 0.813 ±0.042 | 0.719 ±0.047 | 0.551 ±0.057 | 0.342 ±0.061 | **0.119 ±0.029** |
| 3 | 1.00E-06 | 0.931 ±0.026 | 0.920 ±0.022 | 0.904 ±0.019 | 0.869 ±0.026 | 0.815 ±0.044 | 0.705 ±0.050 | 0.549 ±0.052 | 0.328 ±0.053 | 0.099 ±0.046 |
| 1 | 5.00E-05 | 0.937 ±0.023 | 0.914 ±0.020 | 0.886 ±0.025 | 0.833 ±0.040 | 0.744 ±0.048 | 0.612 ±0.065 | 0.449 ±0.083 | 0.250 ±0.040 | 0.068 ±0.022 |
| 2 | 5.00E-05 | 0.933 ±0.021 | 0.918 ±0.022 | 0.894 ±0.022 | 0.869 ±0.026 | 0.820 ±0.032 | 0.699 ±0.051 | 0.533 ±0.074 | 0.299 ±0.065 | 0.090 ±0.020 |
| 3 | 5.00E-05 | 0.924 ±0.021 | 0.915 ±0.021 | 0.897 ±0.016 | 0.864 ±0.025 | 0.806 ±0.038 | 0.710 ±0.052 | 0.549 ±0.040 | 0.334 ±0.050 | 0.096 ±0.030 |
| 1 | 1.00E-05 | 0.935 ±0.024 | 0.915 ±0.025 | 0.886 ±0.025 | 0.839 ±0.035 | 0.750 ±0.050 | 0.635 ±0.059 | 0.472 ±0.068 | 0.271 ±0.036 | 0.083 ±0.021 |
| 2 | 1.00E-05 | 0.935 ±0.023 | 0.919 ±0.026 | 0.895 ±0.021 | 0.869 ±0.029 | 0.795 ±0.043 | 0.685 ±0.052 | 0.536 ±0.050 | 0.313 ±0.045 | 0.095 ±0.016 |
| 3 | 1.00E-05 | 0.937 ±0.026 | **0.930 ±0.024** | **0.912 ±0.031** | **0.884 ±0.023** | 0.817 ±0.045 | 0.718 ±0.052 | 0.541 ±0.074 | 0.317 ±0.064 | 0.098 ±0.024 |
| 1 | 5.00E-06 | **0.942 ±0.022** | 0.929 ±0.017 | 0.895 ±0.021 | 0.854 ±0.025 | 0.768 ±0.046 | 0.645 ±0.068 | 0.475 ±0.065 | 0.275 ±0.043 | 0.075 ±0.027 |
| 2 | 5.00E-06 | 0.931 ±0.026 | 0.918 ±0.025 | 0.901 ±0.018 | 0.870 ±0.020 | 0.797 ±0.041 | 0.682 ±0.054 | 0.510 ±0.049 | 0.313 ±0.069 | 0.090 ±0.025 |
| 3 | 5.00E-06 | 0.932 ±0.028 | 0.917 ±0.027 | 0.902 ±0.019 | 0.865 ±0.032 | 0.822 ±0.042 | 0.712 ±0.044 | 0.534 ±0.046 | 0.298 ±0.045 | 0.086 ±0.023 |
| 1 | 1.00E-06 | 0.937 ±0.019 | 0.919 ±0.015 | 0.889 ±0.025 | 0.841 ±0.036 | 0.751 ±0.054 | 0.624 ±0.058 | 0.469 ±0.066 | 0.279 ±0.051 | 0.073 ±0.024 |
| 2 | 1.00E-06 | 0.925 ±0.028 | 0.913 ±0.030 | 0.888 ±0.027 | 0.849 ±0.031 | 0.790 ±0.039 | 0.672 ±0.055 | 0.499 ±0.072 | 0.289 ±0.050 | 0.079 ±0.028 |
| 3 | 1.00E-06 | 0.931 ±0.022 | 0.918 ±0.019 | 0.897 ±0.017 | 0.863 ±0.023 | 0.792 ±0.038 | 0.668 ±0.029 | 0.526 ±0.047 | 0.296 ±0.042 | 0.079 ±0.031 |
| 1 | 5.00E-05 | 0.934 ±0.023 | 0.916 ±0.023 | 0.889 ±0.026 | 0.846 ±0.033 | 0.768 ±0.059 | 0.653 ±0.054 | 0.496 ±0.075 | 0.289 ±0.060 | 0.082 ±0.022 |
| 2 | 5.00E-05 | 0.935 ±0.027 | 0.918 ±0.025 | 0.900 ±0.025 | 0.865 ±0.032 | 0.801 ±0.036 | 0.690 ±0.035 | 0.532 ±0.063 | 0.313 ±0.055 | 0.085 ±0.020 |
| 3 | 5.00E-05 | 0.929 ±0.021 | 0.911 ±0.022 | 0.895 ±0.023 | 0.854 ±0.028 | 0.804 ±0.032 | 0.684 ±0.045 | 0.542 ±0.046 | 0.312 ±0.055 | 0.090 ±0.027 |
| 1 | 1.00E-05 | 0.939 ±0.022 | 0.922 ±0.026 | 0.900 ±0.026 | 0.853 ±0.032 | 0.797 ±0.042 | 0.692 ±0.049 | 0.525 ±0.085 | 0.312 ±0.054 | 0.115 ±0.027 |
| 2 | 1.00E-05 | 0.928 ±0.028 | 0.915 ±0.027 | 0.897 ±0.024 | 0.862 ±0.031 | 0.793 ±0.039 | 0.684 ±0.061 | 0.510 ±0.061 | 0.299 ±0.039 | 0.081 ±0.022 |
| 3 | 1.00E-05 | 0.934 ±0.024 | 0.924 ±0.024 | 0.902 ±0.018 | 0.874 ±0.025 | 0.819 ±0.040 | 0.720 ±0.054 | 0.550 ±0.066 | 0.320 ±0.064 | 0.097 ±0.029 |
| 1 | 5.00E-06 | 0.938 ±0.024 | 0.922 ±0.024 | 0.896 ±0.024 | 0.848 ±0.042 | 0.767 ±0.045 | 0.665 ±0.040 | 0.491 ±0.046 | 0.266 ±0.037 | 0.075 ±0.023 |
| 2 | 5.00E-06 | 0.935 ±0.021 | 0.922 ±0.020 | 0.902 ±0.021 | 0.872 ±0.030 | 0.812 ±0.038 | 0.692 ±0.054 | 0.531 ±0.055 | 0.305 ±0.049 | 0.082 ±0.035 |
| 3 | 5.00E-06 | 0.931 ±0.025 | 0.918 ±0.025 | 0.900 ±0.026 | 0.866 ±0.028 | 0.811 ±0.037 | 0.711 ±0.045 | 0.528 ±0.077 | 0.309 ±0.058 | 0.083 ±0.031 |
| 1 | 1.00E-06 | 0.937 ±0.031 | 0.917 ±0.033 | 0.882 ±0.032 | 0.822 ±0.044 | 0.736 ±0.059 | 0.617 ±0.067 | 0.467 ±0.074 | 0.250 ±0.058 | 0.063 ±0.016 |
| 2 | 1.00E-06 | 0.929 ±0.026 | 0.916 ±0.025 | 0.900 ±0.025 | 0.863 ±0.030 | 0.812 ±0.029 | 0.706 ±0.045 | 0.543 ±0.056 | 0.313 ±0.051 | 0.089 ±0.025 |
| 3 | 1.00E-06 | 0.931 ±0.020 | 0.917 ±0.020 | 0.898 ±0.020 | 0.869 ±0.033 | 0.812 ±0.031 | 0.697 ±0.040 | 0.537 ±0.068 | 0.311 ±0.050 | 0.077 ±0.033 |

S13 Table: Conventional F1-scores for the D4 validation set.

Scores in cells correspond to average conventional F1 +/- standard deviation (n=10 images, N=1 experiment) and τ_1_ refers to the intersection over union threshold above which predictions are considered true positives (best model per τ_1_ in bold). The purple shaded row corresponds to the model we selected for testing and the purple square in S7b Fig. List of abbreviations: learning rate (Lr).
