# Supplementary material for: aiSEGcell: User-friendly deep learning-based segmentation of nuclei in transmitted light images
Source: PLoS Comput Biol. 2024 Aug 23;20(8):e1012361. doi: 10.1371/journal.pcbi.1012361 (PMC11343410; doi:10.1371/journal.pcbi.1012361)
Supplement: S15 Table — (DOCX) [file pcbi.1012361.s031.docx]

| **Feature** | **Implementation** | **Nucleus** | **Cell** | **Description** |
| --- | --- | --- | --- | --- |
| Area | histomicstk.features.compute_nuclei_features | y | y | Number of pixels the object occupies. |
| Solidity | histomicstk.features.compute_nuclei_features | y | y | A measure of convexity computed as the ratio of the number of pixels in the object to that of its convex hull. |
| Intensity s.d. | histomicstk.features.compute_nuclei_features | y | y | Standard deviation of the intensities of object pixels. |
| Extent | histomicstk.features.compute_nuclei_features | y | y | Ratio of area of the object to its axis-aligned bounding box. |
| Gradient magnitude s.d. | histomicstk.features.compute_nuclei_features | y | y | Standard deviation of gradient data. |
| Eccentricity | histomicstk.features.compute_nuclei_features | y | y | A measure of aspect ratio computed to be the eccentricity of the ellipse that has the same second-moments as the object region. Eccentricity of an ellipse is the ratio of the focal distance (distance between focal points) over the major axis length. The value is in the interval [0, 1). When it is 0, the ellipse becomes a circle. |
| Intensity skewness | histomicstk.features.compute_nuclei_features | y | y | Skewness of the intensities of object pixels. Value is 0 when all intensity values are equal. |
| Distance to closest nucleus | scipy.spatial.distance_matrix | y | n | Distance to the closest nucleus. |
| Gradient magnitude skewness | histomicstk.features.compute_nuclei_features | y | y | Skewness of gradient data. Value is 0 when all values are equal. |
| Focus | skimage.measure.blur_effect | n | y | Metric that indicates the strength of blur in an image (0 for no blur, 1 for maximal blur). |
| Border heterogeneity | custom | y | y | Variance of intensity in contour of object. |
| Multinucleated | custom | n | y | 1 if number of nucleus masks within the cell mask is >1, else 0. |

S15 Table: Morphology and intensity features.
