# Supplementary material for: aiSEGcell: User-friendly deep learning-based segmentation of nuclei in transmitted light images
Source: PLoS Comput Biol. 2024 Aug 23;20(8):e1012361. doi: 10.1371/journal.pcbi.1012361 (PMC11343410; doi:10.1371/journal.pcbi.1012361)
Supplement: S17 Table — (DOCX) [file pcbi.1012361.s033.docx]

| **Antibody** | **Producer** | **Catalogue number** | **Clone** |
| --- | --- | --- | --- |
| CD43 (biotinylated) | invitrogen, eBioscience | 13-0431-85 | eBioR2/60 |
| CD3epsilon (biotinylated) | invitrogen, eBioscience | 13-0031-86 | 145-2C11 |
| CD19 (biotinylated) | invitrogen | 13-0191-86 | eBio1D3 |
| CD19 (biotinylated) | Biolegend | 101504 | MB19-1 |
| NK1.1 (biotinylated) | eBioscience | 13-5941-85 | PK136 |
| TER-119 (biotinylated) | invitrogen, eBioscience | 13-5921-85 | TER-119 |
| B220 (biotinylated) | invitrogen, eBioscience | 13-0452-86 | RA3-6B2 |
| Ly-6G (biotinylated) | invitrogen, eBioscience | 13-5931-86 | RB6-8C5 |
| CD11b (biotinylated) | invitrogen, eBioscience | 13-0112-86 | M1/70 |
| CD41 (biotinylated) | invitrogen, eBioscience | 13-0411-82 | eBioMWReg30 |
| CD16/32-PerCy-Cy5.5 | Biolegend, eBioscience | 101324, 45-0161-82 | 93 |
| CD16/32-APC-Cy7 | Biolegend | 101328 | 93 |
| CD115-BV421 | Biolegend | 135513 | AFS98 |
| Sca1-BV510 | Biolegend | 108129 | D7 |
| Sca1-BV711 | Biolegend | 108131 | D7 |
| Sca1-PacBlue | Biolegend | 108120 | D7 |
| Sca1-PerCP-Cy5.5 | eBioscience | 45-5981-82 | D7 |
| streptavidin-BV570 | Biolegend | 405227 |  |
| streptavidin-BV650 | BD Biosciences | 563855 |  |
| streptavidin-BV711 | BD Biosciences | 563262 |  |
| streptavidin-APC-eFl780 | eBioscience | 47-4317-82 |  |
| cKit-PE-Cy7 | eBioscience | 25-1171-82 | 2B8 |
| cKit-BV711 | BD Biosciences | 563160 | 2B8 |
| cKit-BV510 | Biolegend | 105839 | 2B8 |
| CD34-eFl450 | eBioscience | 48-0341-82 | RAM34 |
| CD34-eFl660 | invitrogen, eBioscience | 50-0341-82 | RAM34 |
| Ly6C-BV510 | Biolegend | 128033 | HK1.4 |
| Ly6C-APC | Biolegend | 128016 | HK1.4 |
| Ly6C-APC/Fire 750 | Biolegend | 128046 | HK1.4 |
| CD135-PE-CF594 | BD Biosciences | 562537 | A2F10 |
| CD135-APC | Biolegend | 135310 | A2F10 |
| CD135-PerCp-eFl710 | eBioscience | 46-1351-82 | A2F10 |
| CD150-PE | Biolegend | 115904 | TC15-12F12.2 |
| CD150-BV650 | Biolegend | 115931 | TC15-12F12.2 |
| CD48-FITC | Biolegend | 103404 | HM48-1 |
| CD48-APCeFl780 | invitrogen | 47-0481-82 | HM48-1 |
| CD11b | Biolegend | 101257 | M1/70 |
| CD41-APC | eBioscience | 17-0411-82 | eBioMWReg30 |
| CD41-PerCp-eFl710 | eBioscience | 46-0411-82 | eBioMWReg30 |
| CD105-eFl450 | eBioscience | 48-1057-42 | SN6 |
| CD105-APC/Fire 750 | Biolegend | 120426 | MJ7/18 |

S17 Table: List of Antibodies.
