# Supplementary material for: aiSEGcell: User-friendly deep learning-based segmentation of nuclei in transmitted light images
Source: PLoS Comput Biol. 2024 Aug 23;20(8):e1012361. doi: 10.1371/journal.pcbi.1012361 (PMC11343410; doi:10.1371/journal.pcbi.1012361)
Supplement: Attachment — Submitted filename: Schirmacher_PBPR_240624.docx [file pcbi.1012361.s034.docx]

**Editor**

*Dear Prof. Dr. Schroeder,*

*Thank you very much for submitting your manuscript "aiSEGcell: user-friendly deep learning-based segmentation of nuclei in transmitted light images" for consideration at PLOS Computational Biology.*

*As with all papers reviewed by the journal, your manuscript was reviewed by members of the editorial board and by several independent reviewers. In light of the reviews (below this email), we would like to invite the resubmission of a significantly-revised version that takes into account the reviewers' comments.*

*Dear Professor Schroeder,*

*Thank you for submitting your work to PLOS Computational Biology. The two referees that have looked at your manuscript agree that the paper presents valuable contributions for the community. However, they also agree that there are some issues that need to be address. Specifically, two major issues need your attention in a revised version of the manuscript: 1) the evaluation metric used should be consistent with previous literature, and should be expanded in detail across datasets and other choices.*
Thank you for your time and interest!
As requested, we now provide our adapted version of the F1-score with multiple thresholds along with the literature-conform (conventional) F1-score (revised Supplementary Table 1-3, 5-14). We adapted the Materials and Methods section “F1 computation” to clarify and motivate the use of both metrics, with more information in revised Supplementary Fig. 16. Furthermore, we adapted the Materials and Methods section “aiSEGcell performance evaluation” to clarify why the object-wise intersection over union (IOU) helps evaluate the co-culture setting of data set D3.
In short, observations based on the adapted and the conventional F1-score generally align, suggesting that both metrics are suitable for model selection. However, we observe that most segmentation errors are inaccurately segmented nuclei counted as both, false positive and false negative, leading the conventional F1-score to underestimate the segmentation quality (revised Supplementary Figure 16). Therefore, we consider our adapted version of the F1-score a more appropriate representation of the observed segmentation quality in our experiments.
Irrespective of the used F1-score, our quantification of the very challenging single cell signaling dynamics that depend on very accurate nucleus segmentations further validates aiSEGcell segmentation quality (Fig. 2).
Finally, while F1-scores help assess segmentation errors for the co-culture setting of D3 (revised Supplementary Table 9-11), we want to highlight the need for an additional analysis approach. Only a subset of cells in D3 images are the murine Megakaryocytes (mMEG) relevant to the analysis. Consequently, an object-wise analysis based on IOU is better suited to quantify the segmentation quality of mMEG than an image-wise metric (i.e. F1-score) that confounds the segmentation quality of different cell types in an image.
IOU and F1-scores now comprehensively describe the segmentation quality on D3.

*2) A comparative evaluation with existing methods such as Cellpose or microSAM would be necessary to understand benefits and challenges of the proposed software.*
Thank you for the suggestion.
While we chose U-Net for its versatility and generally good performance, we do not want to claim that other architectures may not be suitable alternatives. However, to our knowledge, existing software does not provide reliable capabilities to segment nuclei in bright field. We now compared pretrained Cellpose (“nuclei”, “yeast_BF_cp3”) and StarDist (“2D_versatile_fluo”, “2D_versatile_he”) models to aiSEGcell and systematically tested different post-processing settings (revised Supplementary Tables 1-2). Additionally, we explicitly address the comparison in results section 1 “Segmenting nuclei in common bright field images” and add the respective methods section “Cellpose and StarDist pretrained model evaluation”.
We find that available Cellpose and StarDist models are not suitable alternatives to the specialized BF nucleus segmentation of aiSEGcell.

*Please, see the details of the reviewer's comments below. Please, let me know if you have any questions.*

*Best,*

*Juan C. Caicedo*

*We cannot make any decision about publication until we have seen the revised manuscript and your response to the reviewers' comments. Your revised manuscript is also likely to be sent to reviewers for further evaluation.*

*When you are ready to resubmit, please upload the following:*

*[1] A letter containing a detailed list of your responses to the review comments and a description of the changes you have made in the manuscript. Please note while forming your response, if your article is accepted, you may have the opportunity to make the peer review history publicly available. The record will include editor decision letters (with reviews) and your responses to reviewer comments. If eligible, we will contact you to opt in or out.*

*[2] Two versions of the revised manuscript: one with either highlights or tracked changes denoting where the text has been changed; the other a clean version (uploaded as the manuscript file).*

*Important additional instructions are given below your reviewer comments.*

*Please prepare and submit your revised manuscript within 60 days. If you anticipate any delay, please let us know the expected resubmission date by replying to this email. Please note that revised manuscripts received after the 60-day due date may require evaluation and peer review similar to newly submitted manuscripts.*

*Thank you again for your submission. We hope that our editorial process has been constructive so far, and we welcome your feedback at any time. Please don't hesitate to contact us if you have any questions or comments.*

*Sincerely,*

*Juan Caicedo*

*Guest Editor*

*PLOS Computational Biology*

*Daniel Beard*

*Section Editor*

*Have the authors made all data and (if applicable) computational code underlying the findings in their manuscript fully available?*

*The PLOS Data policy requires authors to make all data and code underlying the findings described in their manuscript fully available without restriction, with rare exception (please refer to the Data Availability Statement in the manuscript PDF file). The data and code should be provided as part of the manuscript or its supporting information, or deposited to a public repository. For example, in addition to summary statistics, the data points behind means, medians and variance measures should be available. If there are restrictions on publicly sharing data or code —e.g. participant privacy or use of data from a third party—those must be specified.*

*Reviewer #1: No: Dataset is not yet available, code is available.*

*Reviewer #2: No: See comments on the dataset in the main review.*

As previously discussed, the code and data used to generate all figures/tables and the very large image data set (~70 GB) will be made publicly available with publication. All data has already been uploaded to the ETH Zurich’s certified FAIR repository. We are happy to already provide the reviewers with the private access link in case they want to review it:
<https://libdrive.ethz.ch/index.php/s/BcHDzvksGGL7kkP>
password: xRkf4Tuuz7Uk

*PLOS authors have the option to publish the peer review history of their article (what does this mean?). If published, this will include your full peer review and any attached files.*

*If you choose “no”, your identity will remain anonymous but your review may still be made public.*

*Do you want your identity to be public for this peer review? For information about this choice, including consent withdrawal, please see our Privacy Policy.*

*Reviewer #1: No*

*Reviewer #2: Yes: Constantin Pape*

*Figure Files:*

*While revising your submission, please upload your figure files to the Preflight Analysis and Conversion Engine (PACE) digital diagnostic tool, https://pacev2.apexcovantage.com. PACE helps ensure that figures meet PLOS requirements. To use PACE, you must first register as a user. Then, login and navigate to the UPLOAD tab, where you will find detailed instructions on how to use the tool. If you encounter any issues or have any questions when using PACE, please email us at figures@plos.org.*

*Data Requirements:*

*Please note that, as a condition of publication, PLOS' data policy requires that you make available all data used to draw the conclusions outlined in your manuscript. Data must be deposited in an appropriate repository, included within the body of the manuscript, or uploaded as supporting information. This includes all numerical values that were used to generate graphs, histograms etc.. For an example in PLOS Biology see here: http://www.plosbiology.org/article/info%3Adoi%2F10.1371%2Fjournal.pbio.1001908#s5.*

*Reproducibility:*

*To enhance the reproducibility of your results, we recommend that you deposit your laboratory protocols in protocols.io, where a protocol can be assigned its own identifier (DOI) such that it can be cited independently in the future. Additionally, PLOS ONE offers an option to publish peer-reviewed clinical study protocols. Read more information on sharing protocols at https://plos.org/protocols?utm_medium=editorial-email&utm_source=authorletters&utm_campaign=protocols*

**Reviewer 1**

*Reviewer #1: The paper (will) provide two assets:*

*The dataset of brightfield nuclei (1.1M nuclei - 20K images).*

*A model trained on a subset of this dataset (+napari plugin \ starter code) - already available, repository is well-documented.*

Thank you for your time and suggestions!

*The paper does not propose anything novel in terms of computational methods (training of basic U-Net with in-house dataset) nor does it have interesting choices in the pipelines, besides some questions arise with regard to the evaluation metric and experimental side.*

*While training on Dataset 1 (D1), why no brightness augmentations were used during training?*
Data set 1 (D1) is a large data set (ca. 9000 training images) that is already diverse with respect to its levels of brightness (PBPR Fig. 1a). Indeed, training our model on D1 without brightness augmentations resulted in sufficiently accurate nucleus segmentations to retain even challenging single cell signaling dynamics (Fig. 1-2). In contrast, data sets 3 and 4 (D3 / D4: 24 / 22 training images) are very small and less diverse (PBPR Fig. 1b,c). Therefore, we introduced brightness augmentations to artificially expand the diversity of D3 and D4 training data. We have updated the section “Materials: aiSEGcell model selection” to clarify this point.

PBPR Figure 1: Data set D1 intensity distribution is diverse.

Distribution of image-wise average bright field intensities for data sets (a) D1, (b) D3, (c) D4 (D1 / D3 / D4 n = 8864 / 24 / 22 images).

*How does performance of the model change on the D1 dataset after fine-tuning on D3\D4?*
Thank you for the suggestion. We assume you are referring to a comparison on the data provided in Fig. 1d which is based on dataset D2. Retraining aiSEGcell decreased segmentation performance on the D2 test set (D1 trained / D3 retrained / D4 retrained $\text{F1}_{\text{avg}}^{\text{0.6}}$=0.66 / 0.27 / 0.12; revised Supplementary Table 3). We mention this additional analysis in the results section “aiSEGcell is easily adaptable to new experimental conditions”, noting that a more involved training scheme would potentially retain higher segmentation quality on the D2 test set. However, the training scheme we employ mimics a common user scenario. Using different models for different use cases (D2/D3/D4) is common and viable in practice.

*Evaluation metric: authors use unconventional F1-score with an unconventional fixed threshold (which is not motivated either) and seems like it was completely different to the cited source (where multiple IoU thresholds are used). I would recommend here to fall back to more conventional metrics (or even reconsider the choice of metrics). Two papers came out in Nature Methods that might help you:*

*https://www.nature.com/articles/s41592-023-01942-8*

*https://www.nature.com/articles/s41592-023-02150-0*
As requested, we now provide our adapted version of the F1-score with multiple thresholds along with the literature-conform (conventional) F1-score (revised Supplementary Table 1-3, 5-14). We adapted the Materials and Methods section “F1 computation” to clarify and motivate the use of both metrics, with more information in revised Supplementary Fig. 16. Furthermore, we adapted the Materials and Methods section “aiSEGcell performance evaluation” to clarify why the object-wise intersection over union (IOU) helps evaluate the co-culture setting of data set D3.
In short, observations based on the adapted and the conventional F1-score generally align, suggesting that both metrics are suitable for model selection. However, we observe that most segmentation errors are inaccurately segmented nuclei counted as both, false positive and false negative, leading the conventional F1-score to underestimate the segmentation quality (revised Supplementary Figure 16). Therefore, we consider our adapted version of the F1-score a more appropriate representation of the observed segmentation quality in our experiments.
Irrespective of the used F1-score, our quantification of the very challenging single cell signaling dynamics that depend on very accurate nucleus segmentations further validates aiSEGcell segmentation quality (Fig. 2).
Finally, while F1-scores help assess segmentation errors for the co-culture setting of D3 (revised Supplementary Table 9-11), we want to highlight the need for an additional analysis approach. Only a subset of cells in D3 images are the murine Megakaryocytes (mMEG) relevant to the analysis. Consequently, an object-wise analysis based on IOU is better suited to quantify the segmentation quality of mMEG than an image-wise metric (i.e. F1-score) that confounds the segmentation quality of different cell types in an image.
IOU and F1-scores now comprehensively describe the segmentation quality on D3.

*Did you consider any existing pretrained model to compare your model with?*
Thank you for the suggestion.
While we chose U-Net for its versatility and generally good performance, we do not want to claim that other architectures may not be suitable alternatives. However, to our knowledge, existing software does not provide reliable capabilities to segment nuclei in bright field. We now compared pretrained Cellpose (“nuclei”, “yeast_BF_cp3”) and StarDist (“2D_versatile_fluo”, “2D_versatile_he”) models to aiSEGcell and systematically tested different post-processing settings (revised Supplementary Tables 1-2). Additionally, we explicitly address the comparison in results section 1 “Segmenting nuclei in common bright field images” and add the respective methods section “Cellpose and StarDist pretrained model evaluation”.
We find that available Cellpose and StarDist models are not suitable alternatives to the specialized BF nucleus segmentation of aiSEGcell.

*On a positive side, authors evaluate segmentation w.r.t. downstream biological tasks by training random forest classifiers with morphological features (21) that were extracted from segmented regions.*

*In figure 1d: would be nice to have a threshold (0.6) on the plot and also mean or median value.*
Thank you for the suggestion. Following the notation of the reference suggested above (Hierling et al., Nature Methods 2021) we made the notation of the F1-score more explicit throughout the manuscript. The F1-score notation now always denotes the used threshold and if it is a single image value, average, or aggregated (incl. revised Fig. 1d).

**Reviewer 2**

*Reviewer #2: The manuscript describes a new tool for nucleus segmentation in bright field microscopy. It is based on the established U-Net architecture and a large dataset that is collected by the authors for this purpose. The manuscript first evaluates the performance of the segmentation network on (a train/test split of) this dataset, then evaluates the performance for a downstream biological application, investigates the transfer to different bright field datasets, demonstrates the utility of the method by using the now available fluorescent channel for different readouts and finally describes the tool that makes the method available via CLI and napari.*

*Overall the manuscript is well written and the description of methods and experiments is sound. The software seems to be well documented and relatively easy to use (I did not have time to closely review it).*

*A reliable method for nucleus segmentation in bright field images is a valuable contribution.*

Thank you for your time, your insightful questions, and your helpful suggestions.

*However, two points should be addressed before accepting the manuscript:*

*1. The method description for the nucleus segmentation is not sufficient. While the authors describe the U-Net architecture they do not describe at all how an instance segmentation is obtained from the predictions of the U-Net.*

*My best guess given the description given is that the U-Net only predicts a single channel for foreground and then thresholding and connected components are applied. If this is indeed the case, then this work has a major drawback: it will likely not work well for images with a high-confluency where nuclei can be almost touching and will thus be likely merged incorrectly into a single object. (And this fact is the reason for the complex post-processing procedures in StarDist, CellPose, etc.).*

*To address this weakness:*

*- Please describe the post-processing of the network outputs in detail.*

*- If my assumption about the simple post-processing is correct please address the case of images with high confluency:*

*-- Are such images part of the training / testing data? Did you explicitly check for incorrect merges for images with high confluency?*

*-- If this is indeed a problem: I would suggest to address this issue by including a slightly more complex network objective and post-processing, e.g. predicting both foreground and boundaries between nuclei, and adapting the post-processing, to enable separation of close or touching objects.*
Thank you for the suggestion. We now clarified the post-processing of the network output in Materials and Methods section “Image segmentation”. Indeed, we use connected components on a single foreground channel with a threshold to obtain instance segmentations.

To address your point regarding higher cell densities, we now acquired new experimental data of densely seeded cells, to assess if confluent images lead to incorrect merges (revised Supplementary Table 16) and added the respective Materials and Methods section “High density experiment (D7)”. The new results show that aiSEGcell also accurately segments nuclei in densely seeded images and that incorrectly merged nuclei are very rare. In fact, incorrect merges occur with the same frequency as in images with sparsely seeded cells (revised Supplementary Fig. 3, revised Supplementary Table 4-5). Notably, we did not use the newly acquired data set D7 to (re)train aiSEGcell.

*2. I find the presentation of results for the retraining on new datasets confusing. Figure 3 mixes evaluation of the downstream analysis with qualitative results and a quantitative evaluation of the F1-Score and does so inconsistently across the different new datasets. I would suggest to create a figure or table that compares the F1-Score of your model before and after retraining for all new datasets in a consistent manner. This can either be added to Figure 3 (potentially replacing f and h since this information should be in the new element) or be added to the supplementary material.*
Thank you for the suggestion. As requested, we now provide F1-scores for all Fig. 3 associated analyses (revised Supplementary Table 6, 9-14). Furthermore, we adapted the Materials and Methods section “aiSEGcell performance evaluation” to clarify why the object-wise intersection over union (IOU) helps evaluate the co-culture setting of data set D3.

While F1-scores help assess segmentation errors for the co-culture setting of D3 (revised Supplementary Table 9-11), we want to highlight the need for an additional analysis approach. Only a subset of cells in D3 images are the murine Megakaryocytes (mMEG) relevant to the analysis. Consequently, an object-wise analysis based on IOU is better suited to quantify the segmentation quality of mMEG than an image-wise metric (i.e. F1-score) that confounds the segmentation quality of different cell types in an image.
IOU and F1-scores now comprehensively describe the segmentation quality on D3.

*Besides these two points I have a few minor comments and suggestions.*

*- A comparison to CellPose would be helpful to understand the practical impact the method has over existing methods. For this you could compare application to one of the new datasets, ideally even with fine-tuning of CellPose. (Such experiments always have many degrees of freedom, so I will not make a recommendation for acceptance dependend on it. Nevertheless, I think an accurately reported comparison would be helpful for the the reader.)*
Thank you for the suggestion.
While we chose U-Net for its versatility and generally good performance, we do not want to claim that other architectures may not be suitable alternatives. However, to our knowledge, existing software does not provide reliable capabilities to segment nuclei in bright field. We now compared pretrained Cellpose (“nuclei”, “yeast_BF_cp3”) and StarDist (“2D_versatile_fluo”, “2D_versatile_he”) models to aiSEGcell and systematically tested different post-processing settings (revised Supplementary Tables 1-2). Additionally, we explicitly address the comparison in results section 1 “Segmenting nuclei in common bright field images” and add the respective methods section “Cellpose and StarDist pretrained model evaluation”.
We find that available Cellpose and StarDist models are not suitable alternatives to the specialized BF nucleus segmentation of aiSEGcell.

*- The FastER used for segmentation of the fluorescence channel could be briefly explained in "Materials: Image Segmentation". How does it work, how reliable was it (qualitatively).*
Thank you. We have updated the section “Materials: Image segmentation” to briefly describe the fastER methodology and its reliability.

*- Since the data is not yet available I could not review the quality or documentation of the dataset. Since this dataset is one of the main contributions of this publication that would have been desirable. However, the authors give a clear plan on how they will make the data available, so I will not make my recommendation available on it. As a sidenote:is there a particular reason to not go for a more centralized solution like BioImageArchive (or similar EBI repository) or zenodo? I personally think these solutions are better suited to FAIR data access than institute specific solutions. (To be clear, this is not relevant for my recommendation.)*
Thank you for your comment. Beyond the software, this manuscript provides 2 pretrained models, the data sets, and the raw data and analysis scripts to replicate all figures and tables. Zenodo and other general-purpose FAIR repositories have a storage limit of 50 GB which does not suffice in our case (~70 GB image data), or only allow image data (BIA) but not the other data we make available. In contrast, the ETH Research collection is a general-purpose FAIR repository (accredited FAIR repository of a federal Swiss university, so long-term reliability / availability isn’t an issue) that has no storage or file type limits. Having all data in one place will make it easier and more straight forward for users, it was therefore better suited for our use-case.
